# β-Coronaviruses exploit ESCRT for virion assembly and egress

**DOI:** 10.1128/mbio.00979-25

**Published:** 2025-05-23

**Authors:** Yuanyuan Zhang, Linlong Huang, Chaoqi Ren, Weiyang Wang, Xinlu Wang, Guangxia Gao

**Affiliations:** 1 Key Laboratory of Biomacromolecules (CAS), CAS Center for Excellence in Biomacromolecules, Institute of Biophysics, Chinese Academy of Sciences, Beijing, China; 2 University of Chinese Academy of Sciences 555047, Beijing, China; Johns Hopkins University, Baltimore, Maryland, USA

**Keywords:** β-coronaviruses, assembly and egress, ESCRT components, TSG101, VPS28

## Abstract

β-Coronaviruses assemble at the endoplasmic reticulum-to-Golgi compartment and exit cells through the lysosomal trafficking pathway. However, the molecular mechanisms of virion assembly and egress are largely unknown. Here, we report that β-coronaviruses recruit endosomal sorting complexes required for transport (ESCRTs) to facilitate virion assembly and egress. The viral proteins N and M interacted with ESCRT components TSG101 and VPS28, respectively. Electron microscopy analysis revealed that virion assembly was disrupted by TSG101 knockdown at an early stage and by VPS28 knockdown at a late stage. Knockdown of ESCRT components MVB12A and CHMP6 did not affect virion assembly but inhibited virion egress. Downregulation of these ESCRT factors or VPS4A, or treatment of cells with the TSG101 antagonist tenatoprazole significantly inhibited the production of the virion-like particles of β-coronaviruses and the replication of human coronavirus OC43. These findings indicate that β-coronaviruses exploit ESCRT for virion assembly and egress and suggest that the interaction interface between ESCRT and the viruses could be a target for the development of broad-spectrum anti-coronavirus therapeutics.

**IMPORTANCE:**

β-Coronaviruses have caused disastrous pandemics and may cause serious pandemics in the future. Virion assembly and egress are critical steps in the life cycle of coronaviruses. However, despite extensive studies in the past few years, the molecular mechanisms for virion assembly and egress are still largely unknown. Here we show that β-coronaviruses recruit ESCRT components TSG101 and VPS28 for virion assembly and that ESCRT components MVB12A and CHMP6 are required for virion egress. Treatment of cells with the TSG101 antagonist inhibited the assembly of multiple β-coronaviruses. These findings indicate that ESCRT participates in β-coronavirus assembly and egress and might be a potential target for the development of broad-spectrum anti-coronavirus therapeutics.

## INTRODUCTION

Human β-coronaviruses, such as severe acute respiratory syndrome coronavirus 2 (SARS-CoV-2), severe acute respiratory syndrome coronavirus, human coronavirus OC43 (HCoV-OC43), Middle East respiratory syndrome coronavirus (MERS-CoV), and human coronavirus HKU1 (HCoV-HKU1), are enveloped particles containing a positive sense, single-stranded genomic RNA (1). The 5′ two-thirds of the genome encodes non-structural proteins that are mostly involved in viral RNA replication and transcription (2, 3). The 3′ one-third of the genome encodes structural proteins and accessory proteins (2). The four structural proteins, spike (S), membrane (M), envelope (E), and nucleocapsid (N), are the core constituents of the virion particle (3, 4). Co-expression of the four structural proteins is sufficient to produce virion-like particles (VLPs) (5, 6). Co-expression of M and N is the minimal requirement for VLP formation (7, 8). M is critical for virus assembly and egress during virus morphogenesis (9, 10). N binds to the viral genome and interacts with M to assist virus assembly (11, 12). The E protein forms ion channels in the viral membrane and participates in virion assembly (13). The S protein is essential for viral entry through binding to the cell surface receptor and initiating fusion with the host cell membrane (14, 15). Structural proteins and the associated viral genomic RNA assemble and bud to form new virions at the endoplasmic reticulum-to-Golgi compartment (16, 17). Several lines of evidence suggest that the virions exit cells via the lysosomal trafficking pathway (18, 19). It is believed that the viruses utilize host factors for virion assembly and egress from the producing cells. However, the molecular mechanisms underlying these processes are not clear.

The endosomal sorting complexes required for transport (ESCRTs) are conserved in all eukaryotes and consist of four functionally distinct subcomplexes, ESCRT-0, ESCRT-I, ESCRT-II, and ESCRT-III and several accessory proteins (20). ESCRT functions in many cellular processes such as the formation of multivesicular bodies (MVBs) (21), membrane repair and restoration (22), cell abscission during cytokinesis (23, 24), neuron pruning (25), and exovesicle shedding (26). Recruitment of ESCRT factors to various cellular sites is dependent on ESCRT adaptor proteins. For example, ESCRT adaptor apoptosis-linked gene-2 interacts with ESCRT components for the repair of injured membranes (27).

Multiple viruses have been reported to utilize the ESCRT pathway for virion assembly and release. Retroviruses were the first to be reported to exploit ESCRT for virion budding (28). These viruses usually use short peptide motifs in the viral structural proteins termed “late domain” to recruit ESCRT factors or ESCRT-associated E3 ubiquitin ligases to initiate the process. The three well-characterized late motifs are Pro-Thr/Ser-Ala-Pro (“P[T/S]AP”), Tyr-Pro-Xn-Leu (“YPX_n_L”), wherein X can vary in identity and sequence length), and Pro-Pro-X-Tyr (“PPXY”). The P[T/S]AP motif binds to TSG101, a component of ESCRT-I, and the YPX_n_L motif binds to the ESCRT factor ALIX (29, 30). The PPXY motif usually binds to an E3 ubiquitin ligase of the Nedd4 family to induce the ubiquitination of a viral structural protein to mediate its interaction with ESCRT complexes (31–33). Recruitment of the upstream ESCRT components to the virus budding sites leads to transient recruitment of the late-acting ESCRT-III. In turn, ESCRT-III recruits the ATPase VPS4. As the only known enzyme in the ESCRT pathway, VPS4 drives both membrane fission and recycling of ESCRT-III subunits to their soluble state (34). Subsequent studies indicated that other enveloped viruses, such as herpes simplex virus (35, 36), hepatitis C virus (37, 38), dengue virus (39, 40), and Japanese encephalitis virus (40, 41), also exploit the ESCRT pathway to complete virus replication. Some new “late motifs” were found in the structural proteins of these viruses. In the structural proteins of β-coronaviruses, no obvious late motifs can be identified. It was thus not clear whether β-coronaviruses engage the ESCRT pathway for virion production.

In the present study, we provide evidence showing that β-coronaviruses recruit ESCRT for virion assembly and egress. Downregulation of certain ESCRT components impaired virion production and virus replication.

## RESULTS

### Knockdown of certain ESCRT components impairs β-coronavirus VLP production

To investigate viral assembly and egress of β**-**coronaviruses, we first set up a VLP production system. The coding sequences of N, M, and E were cloned into a eukaryotic expression vector to construct a plasmid expressing N, M, and E (pNEM) (Fig. S1A). In this construct, the coding sequences of N and M are under the transcriptional control of cytomegalovirus (CMV) and EF1α promoters, respectively. The coding sequence of E is downstream of N with its translation initiated by encephalomyocarditis virus internal ribosome entry site (EMCV IRES). A Flag-tag was fused to the N-terminus of M of HCoV-OC43 and HCoV-HKU1 to facilitate detection using Western blotting. Considering that S was not required for VLP formation and that VLPs containing S were not stable in the process of VLP purification (6, 42–45), S was not included. To produce VLP, pNEM was transiently transfected into 293T cells. VLP-containing supernatants were first purified through 20% sucrose ultracentrifugation and then fractionated through sucrose velocity sedimentation centrifugation (Fig. S1A). The distribution of the viral proteins was analyzed by Western blotting. The viral proteins M and N were detected mainly in fractions 6–8, suggesting that they were in particles. Similar sedimentation profiles were observed for all the VLPs tested, including HCoV-OC43, SARS-CoV-2, MERS-CoV, and HCoV-HKU1 (Fig. S1B). The purified HCoV-OC43 VLP was further analyzed by transmission electron microscopy (TEM). The VLP looked very similar to the inactivated virion particles in size and shape (Fig. S1C). Similar results were obtained with SARS-CoV-2 VLP (Fig. S1C).

In an attempt to understand the mechanisms for β-coronavirus assembly and egress, we searched for the host factors that are incorporated into or associated with the VLP or virion particles. The purified VLPs of SARS-CoV-2 and HCoV-OC43 and virion particles of HCoV-OC43 were analyzed by mass spectrometry. TSG101, a key component of ESCRT-I, was reproducibly identified in all these samples (Table S1; Data Set S1). Considering that ESCRT proteins are packaged into extracellular vesicles and can contaminate virion samples, we also analyzed a parallel culture supernatant sample of control cells which did not express any viral proteins. Only ALIX but not TSG101 was detected in the control samples (Table S1; Data Set S1). While these results still could not exclude the possibility that the ESCRT proteins detected in the virion and VLP samples were from the contamination of extracellular vesicles, they suggested that ESCRT might be involved in β-coronavirus production.

To explore whether ESCRT is required for the production of coronavirus VLP, pNEM was transfected into 293T cells along with an siRNA targeting an endogenous ESCRT component. The culture supernatant was collected. HIV-1 VLP, which was produced separately in 293T cells, was added to the coronavirus VLP-containing culture supernatant to serve as a control for sample handling. The VLPs were partially purified through 20% sucrose ultracentrifugation. The relative protein levels of N in the culture supernatant and cell lysate were measured by Western blotting. The relative VLP release ratio was calculated as the protein level of N in the VLP divided by that in the cell lysate. We analyzed the effects of ESCRT component knockdown on VLP production of multiple β-coronaviruses. Knockdown of ESCRT-I components VPS28, MVB12A, VPS37B, and TSG101 (Fig. S2A, B, S3A and B) and ESCRT-III components CHMP1A and CHMP6 (Fig. S2C and S3C) reduced the release ratio of SARS-CoV-2. Given that VPS4A has been reported to play a critical role in virion assembly and release of multiple envelope viruses (38, 46, 47), we tested the effect of VPS4A knockdown on VLP production. Knockdown of VPS4A significantly inhibited the VLP production of SARS-CoV-2 (Fig. S2E and S3E). Knockdown of ESCRT-I components TSG101, VPS28, MVB12A, MVB12B, and VPS37C (Fig. S4A) and ESCRT-III components CHMP2B, CHMP3, CHMP5, and CHMP6 (Fig. S4B) and VPS4A (Fig. S4C) reduced the VLP release ratio of HCoV-OC43. The VLP production of MERS-CoV was significantly reduced by knocking down VPS28, MVB12A, VPS37B, VPS37C, TSG101, CHMP6, and VPS4A (Fig. S5). The VLP production of HCoV-HKU1 was reduced by the downregulation of multiple ESCRT components, the most obvious ones including VPS28, MVB12A, VPS37B, VPS37C, TSG101, CHMP1A, CHMP2A, CHMP3, CHMP5, CHMP6, ALIX, and VPS4A (Fig. S6).

Collectively, the above results indicated that knockdown of certain ESCRT components reduced coronavirus VLP production. For different viruses, VLP production was affected by the knockdown of different components, but some were shared by all the viruses tested, including TSG101, VPS28, MVB12A, CHMP6, and VPS4A (Table S2). We focused on these ESCRT components in the following studies. Noticeably, ESCRT functions as complexes. The protein levels and contribution to the function of ESCRT of the components in cells are expected to be different. Since the depletion of the ESCRT components by siRNA knockdown was not complete, depletion of each component is expected to have different effects on the function of the complex. This would explain why depletion of the components had different effects on virion production.

### TSG101, VPS28, MVB12A, CHMP6, or VPS4A knockdown impairs HCoV-OC43 replication

**Fig 1:**
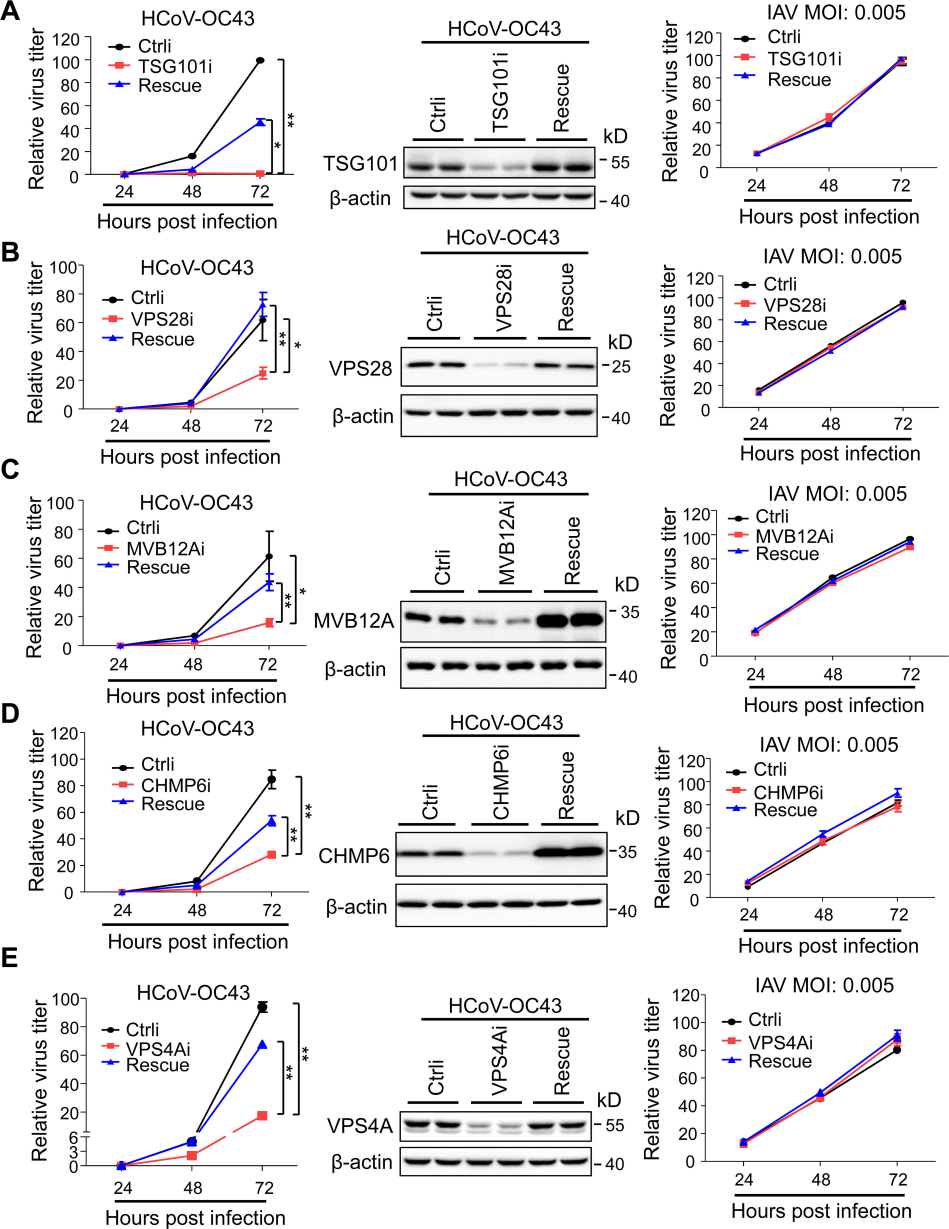
Knockdown of certain ESCRT components impairs HCoV-OC43 replication. (**A–E**) 293T cells were transfected with the siRNA indicated with or without a rescue expression construct. The cells were then infected with HCoV-OC43 or IAV-Gluc. Culture supernatants were collected to measure relative virus titers at the time points indicated. The cells were collected at 72 h post-infection for Western blot analysis. The Western blotting results of the cells infected with IAV and the results of repeated experiments can be found in Fig. S7. Ctrl, control; MOI, multiplicity of infection; R, rescue. * denotes *P* < 0.05 and ** denotes *P* < 0.01.

To determine whether knockdown of these factors inhibits β-coronavirus replication, 293T cells were first transfected with a control siRNA or an siRNA targeting TSG101, VPS28, MVB12A, CHMP6, or VPS4A. To confirm the specificity of the siRNA, a rescue construct expressing the protein was used. The cells were then infected with HCoV-OC43. Viral replication was monitored by measuring the genomic RNA levels in the culture supernatants for 72 h. Knockdown of TSG101, VPS28, MVB12A, CHMP6, and VPS4A all significantly inhibited virus replication, and the virus replication was partially or completely restored when a rescue construct was expressed (Fig. 1; Fig. S7). In contrast, knockdown of these ESCRT components had little effect on the replication of influenza A virus (IAV) (Fig. 1; Fig. S7), which has been reported to replicate in an ESCRT-independent manner (48, 49). These results indicated that the effect of the ESCRT component knockdown on HCoV-OC43 replication was not caused by general toxicity. Similar results were also obtained in A549 cells, a human lung epithelial cell line widely used in the studies of respiratory viruses (Fig. S8).

To further demonstrate that knockdown of the ESCRT components impaired the production of the virion particles, 293T cells were transfected with the siRNA targeting TSG101, VPS28, MVB12A, CHMP6, or VPS4A and then infected with HCoV-OC43. At 24 h post-infection, the viral RNA levels in the culture supernatants and cell lysates were measured. Under this condition, the viral RNA level in the culture supernatant served as an indicator of the amount of the virus released into the culture supernatant, and the viral RNA level in the cell lysate served as an indicator of the virus replication in the cells. As expected, knockdown of TSG101, VPS28, MVB12A, CHMP6, and VPS4A significantly decreased the viral RNA levels in the culture supernatants (Fig. S9). In contrast, knockdown of these ESCRT components had no significant effect on the viral RNA levels in the cell lysates (Fig. S9). These results further show that knockdown of these ESCRT components inhibited virus production.

### TSG101 or VPS28 knockdown inhibits HCoV-OC43 virion assembly, and MVB12A or CHMP6 knockdown inhibits virion egress

**Fig 2:**
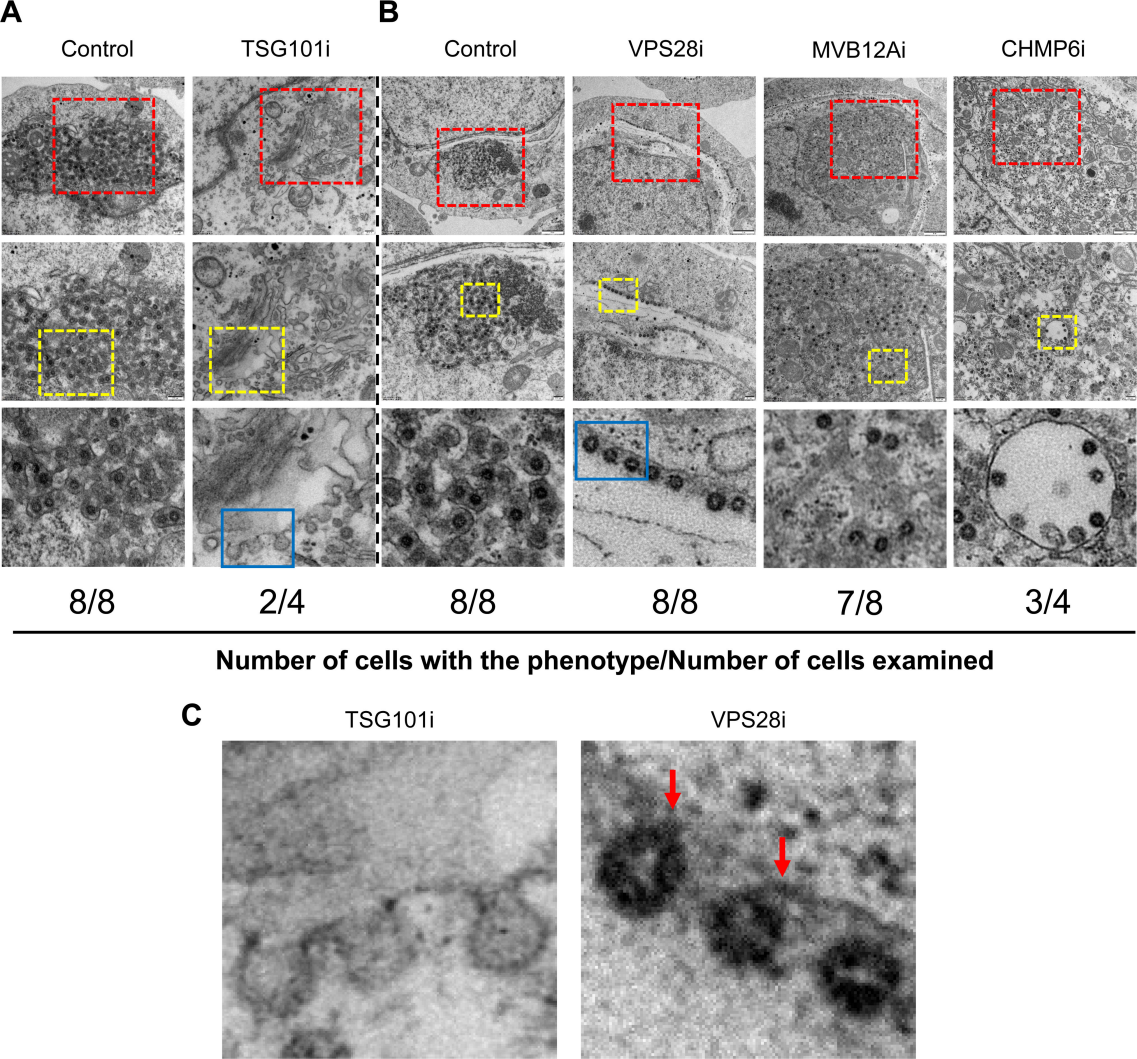
TEM analysis of the effect of ESCRT component knockdown on HCoV-OC43 virion assembly and egress. 293T cells were transfected with the siRNA indicated and infected with HCoV-OC43 (MOI = 0.1) for 2 h. At 48 h post-infection, cells were fixed and analyzed by 100 kV TEM. (**A** and **B**) The images in the middle panels show the enlarged views of the selected area within the red rectangles (magnification: ×49,000 in panel A, ×30,000 in panel B), and the images in the lower panels show the enlarged views of the selected area within the yellow rectangles (magnification: ×110,000 in panel A, ×160,000 in panel B). The scale bars in the upper panels are 200 nm (magnification: ×30,000) in panel **A** and 1 µm (magnification: ×13,000) in panel** B**. (**C**) The images show the enlarged views of the selected area within the blue rectangles (magnification: ×680,000 in TSG101i, ×850,000 in VPS28i). The red arrows indicate the sites where the virions are connected to the membrane.

In an attempt to further understand at which step knockdown of the ESCRT components affects coronavirus production, TEM analysis was employed. TSG101, VPS28, MVB12A, and CHMP6 were knocked down individually in 293T cells, followed by infection of the cells with replication-competent HCoV-OC43. At 48 h post-infection, the cells were subjected to TEM analysis. In the control cells, virion particles were detected in clusters in the cytoplasm (Fig. 2). In TSG101 knockdown cells, much fewer cells containing virion or virion-like particles could be found, consistent with the above results that TSG101 knockdown significantly inhibited viral replication. In some of these cells, a striking observation was that virion assembly was not complete (Fig. 2A). The assembly process was disrupted, leaving the virion-like particles attached to the inner membrane (Fig. 2A and C). In the other cells, the virion particles looked the same as in the control cells. Our explanation is that the siRNA transfection efficiency was not 100%. In some cells, the expression of TSG101 was not downregulated, and in these cells, the virion particles were assembled properly.

In VPS28 knockdown cells, the virion particles appeared to be assembled but attached to the inner membrane (Fig. 2B). However, a close look at the virion particles revealed that the assembly was not fully complete: the virion “circle” was not closed and connected to the membrane (Fig. 2C). In MVB12A knockdown cells, the virion particles looked complete (Fig. 2B), and the virion particles were dispersed in the cytoplasm rather than in clusters (Fig. 2B). In CHMP6 knockdown cells, the assembled virion particles seemed to be trapped in small vesicles in the cytoplasm (Fig. 2B). These results indicated that knockdown of the ESCRT components TSG101, VPS28, MVB12A, and CHMP6 impaired virion assembly and egress (see below for further discussion).

### TSG101 interacts with coronavirus N

**Fig 3:**
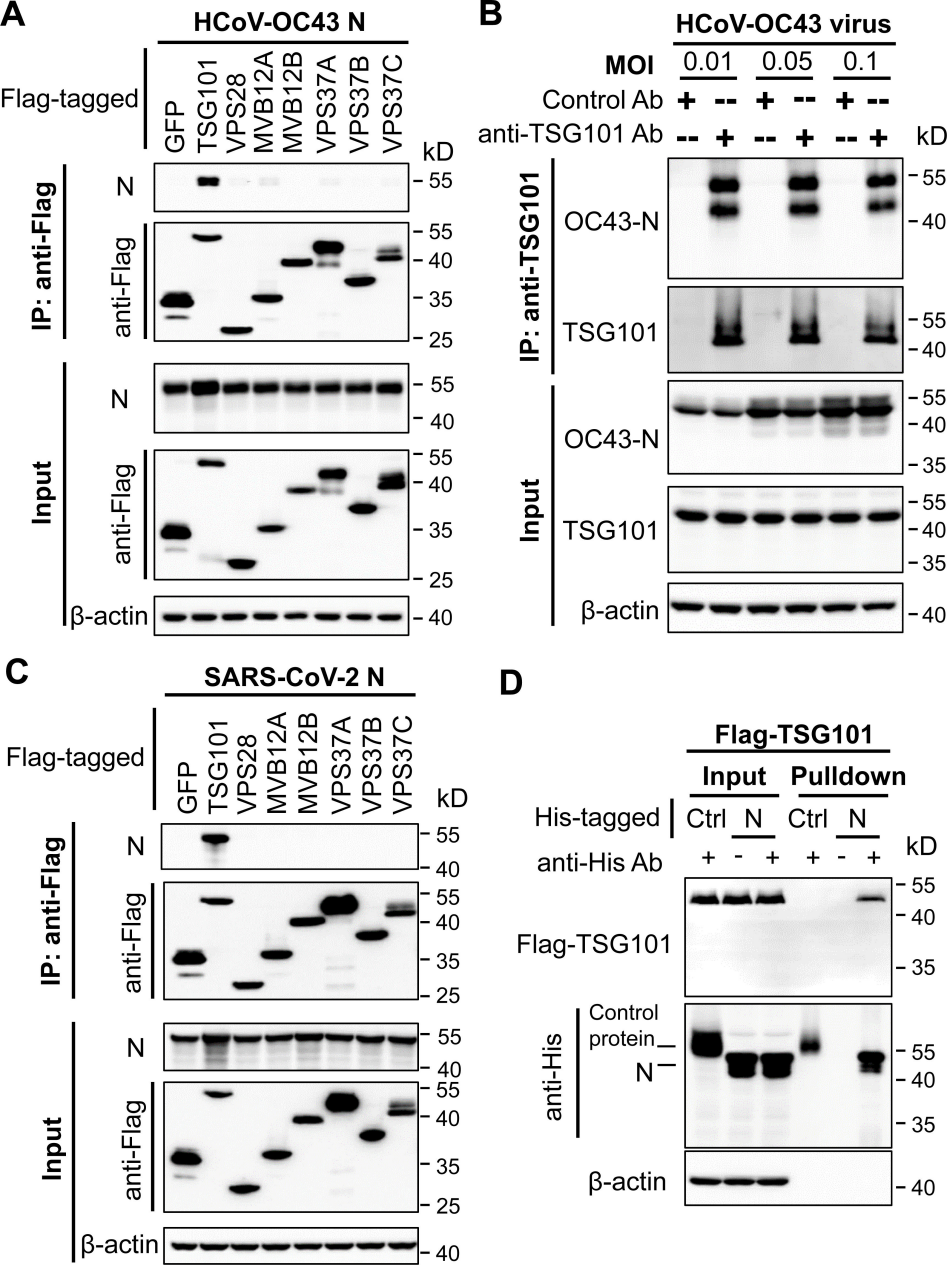
TSG101 interacts with HCoV-OC43 N. (**A**) HCoV-OC43 N protein and the Flag-tagged protein indicated were co-expressed in 293T cells. The cells were lysed; cell lysates were immunoprecipitated with anti-Flag affinity gel and subjected to Western blot analysis using rabbit anti-Flag or anti-HCoV-OC43 N antibody. (**B**) 293T cells were infected with HCoV-OC43 at the MOI indicated. At 48 h post-infection, cells were lysed, and the cell lysate was immunoprecipitated with rabbit anti-TSG101 antibody and subjected to Western blot analysis using rabbit anti-HCoV-OC43 N polyclonal antibody or mouse anti-TSG101 monoclonal antibody. Control antibody: rabbit anti-Flag monoclonal antibody. (**C**) SARS-CoV-2 N interacts with TSG101. The experimental procedure was the same as described above. SARS-CoV-2 N was detected using rabbit anti-SARS-CoV-2 N antibody. (**D**) Purified His-tagged SARS-CoV-2 N recombinant protein was incubated with the cell lysate transiently expressing Flag-TSG101 and immunoprecipitated with anti-His antibody, and the precipitates were analyzed by Western blotting. A His-tagged recombinant protein that does not interact with TSG101 was used as a negative control. Data presented are representative of two independent experiments.

Previous studies suggested that viruses usually recruit ESCRT through interactions of the viral structural proteins with ESCRT components (28). To explore whether structural proteins of β-coronaviruses interact with ESCRT components, the N protein was co-expressed with a subset of Flag-tagged ESCRT proteins and analyzed for their interactions by co-immunoprecipitation assays. Immunoprecipitation of TSG101 co-precipitated the N protein of HCoV-OC43 (Fig. 3A). In contrast, the other ESCRT components failed to interact with N (Fig. 3A). To test whether the endogenous TSG101 interacts with N expressed from virus replication, 293T cells were infected with HCoV-OC43. Immunoprecipitation of endogenous TSG101 co-precipitated N (Fig. 3B).

To test whether TSG101 interacts with the N proteins of other coronaviruses, Flag-tagged TSG101 was co-expressed with the N proteins of SARS-CoV-2 (Fig. 3C), MERS-CoV (Fig. S10A), or HCoV-HKU1 (Fig. S10B), and the interaction was analyzed by coimmunoprecipitation assay. As expected, TSG101 interacted with the N proteins of all these viruses (Fig. 3C; Fig. S10A and B). To further show the interaction of TSG101 with N, bacterially expressed His-tagged SARS-CoV-2 N was incubated with the lysate of 293T cells overexpressing Flag-tagged TSG101. Immunoprecipitation of N pulled down TSG101 (Fig. 3D). Collectively, these results indicated that coronavirus N interacts with TSG101.

### VPS28 interacts with coronavirus M

**Fig 4:**
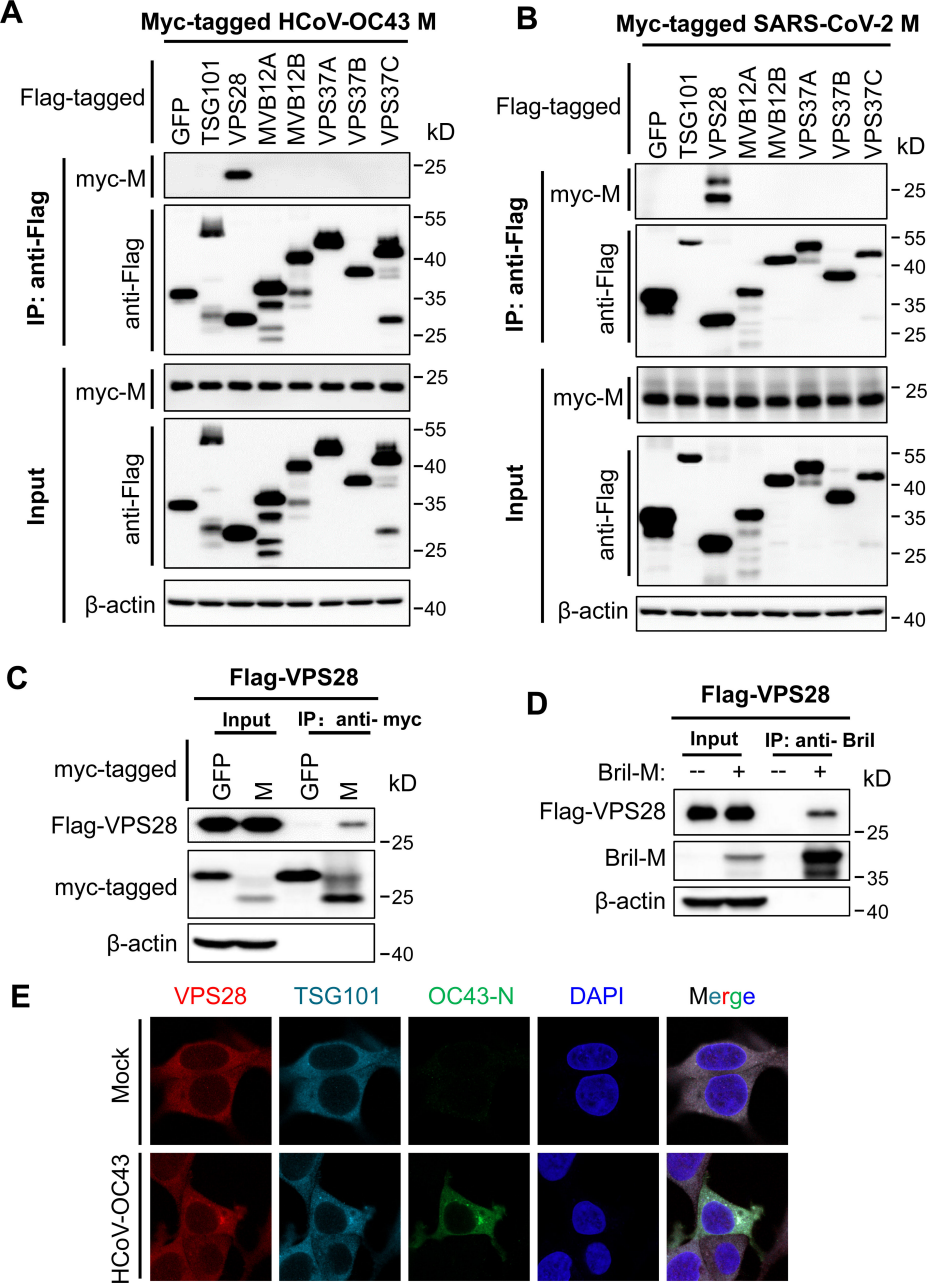
VPS28 interacts with coronavirus M. (A and B) Myc-tagged HCoV-OC43 M (**A**) or SARS-CoV-2 M (**B**) and the Flag-tagged protein indicated were co-expressed in 293T cells. The cell lysate was immunoprecipitated with anti-Flag affinity gel and subjected to Western blot analysis using rabbit anti-Flag or anti-myc antibody. (**C**) Flag-tagged VPS28 and myc-tagged SARS-CoV-2 M were transiently expressed in 293T cells. Myc-tagged green fluorescent protein (GFP) was used as a negative control. The cells were lysed with phosphate-buffered saline (PBS) supplemented with 0.03% n-dodecyl-b-D-maltoside and protease inhibitor cocktail. The cell lysate was immunoprecipitated with anti-myc affinity gel and subjected to Western blotting. (**D**) Purified SARS-CoV-2 M recombinant protein was incubated with the cell lysate expressing Flag-VPS28, pulled down with anti-Bril antibody, and analyzed by Western blotting. (**E**) 293T cells were mock infected or infected with HCoV-OC43. The cells were fixed in paraformaldehyde at 24 h post-infection. VPS28 was stained with mouse anti-VPS28 antibody and Alexa Fluor TRITC-conjugated anti-mouse secondary antibody (red). TSG101 was stained with CoraLite 594-conjugated TSG101 monoclonal antibody (original). HCoV-OC43 N was stained with rabbit anti-N antibody and ABFlo 488-conjugated anti-rabbit secondary antibody (green). Fluorescence images were obtained with confocal fluorescence microscopy. DAPI, 4′,6-diamidino-2-phenylindole; IP, immunoprecipitation.

We next explored whether the M protein interacts with ESCRT components. The myc-tagged M was co-expressed with a subset of Flag-tagged ESCRT proteins, and the interaction was analyzed by coimmunoprecipitation assays. Immunoprecipitation of VPS28 co-precipitated myc-tagged M of HCoV-OC43 (Fig. 4A), SARS-CoV-2 (Fig. 4B), MERS-CoV (Fig. S11A), and HCoV-HKU1 (Fig. S11B). In a reciprocal experiment, immunoprecipitation of SARS-CoV-2 M co-precipitated VPS28 (Fig. 4C). To further show the interaction between VPS28 and M, Bril-tagged SARS-CoV-2 M was bacterially expressed, partially purified, and incubated with the lysate of 293T cells overexpressing Flag-VPS28. Immunoprecipitation of SARS-CoV-2 Bril-M pulled down Flag-VPS28 (Fig. 4D). Similarly, immunoprecipitation of the Bril-M of HCoV-OC43 (Fig. S11C), MERS-CoV (Fig. S11D), and HCoV-HKU1 (Fig. S11E) pulled down Flag-VPS28.

The interactions of VPS28 with coronavirus M were also analyzed by immunostaining assays. SARS-CoV-2 M was expressed in 293T cells, and the localizations of M and endogenous VPS28 were observed by fluorescence microscopy. In the empty vector transfected control cells, both VPS28 and TSG101 were diffusely distributed in the cytoplasm. In the M-expressing cells, VPS28 was relocalized to the paranuclear region (Fig. S12A). TSG101 was also relocalized to this region, likely through TSG101-VPS28 interaction in ESCRT-I. In contrast, in the N-expressing cells, N, TSG101, and VPS28 were diffusely distributed in the cytoplasm; no obvious relocalization of TSG101 or VPS28 was observed (Fig. S12A). The localizations of other coronaviruses, M and endogenous VPS28, were also observed, and similar results were obtained (Fig. S12B).

We next tried to demonstrate the interaction of the endogenous VPS28 with M in virus-infected cells. Due to the lack of an antibody to detect the M protein of HCoV-OC43, we could not directly analyze the co-localization of M with VPS28. We thus analyzed the relocalization of endogenous VPS28 upon HCoV-OC43 infection. The cells were infected with HCoV-OC43, with N protein serving as an indicator of virus infection, and analyzed for the localization of the endogenous VPS28 and TSG101. In the control cells, both VPS28 and TSG101 were diffusely distributed in the cytoplasm (Fig. 4E). While in the infected cells, a fraction of VPS28 and TSG101 relocalized to the paranuclear region and co-localized with N (Fig. 4E). These results further demonstrate the interaction between VPS28 and M.

### TSG101 antagonist tenatoprazole inhibits the production of β-coronaviruses

**Fig 5:**
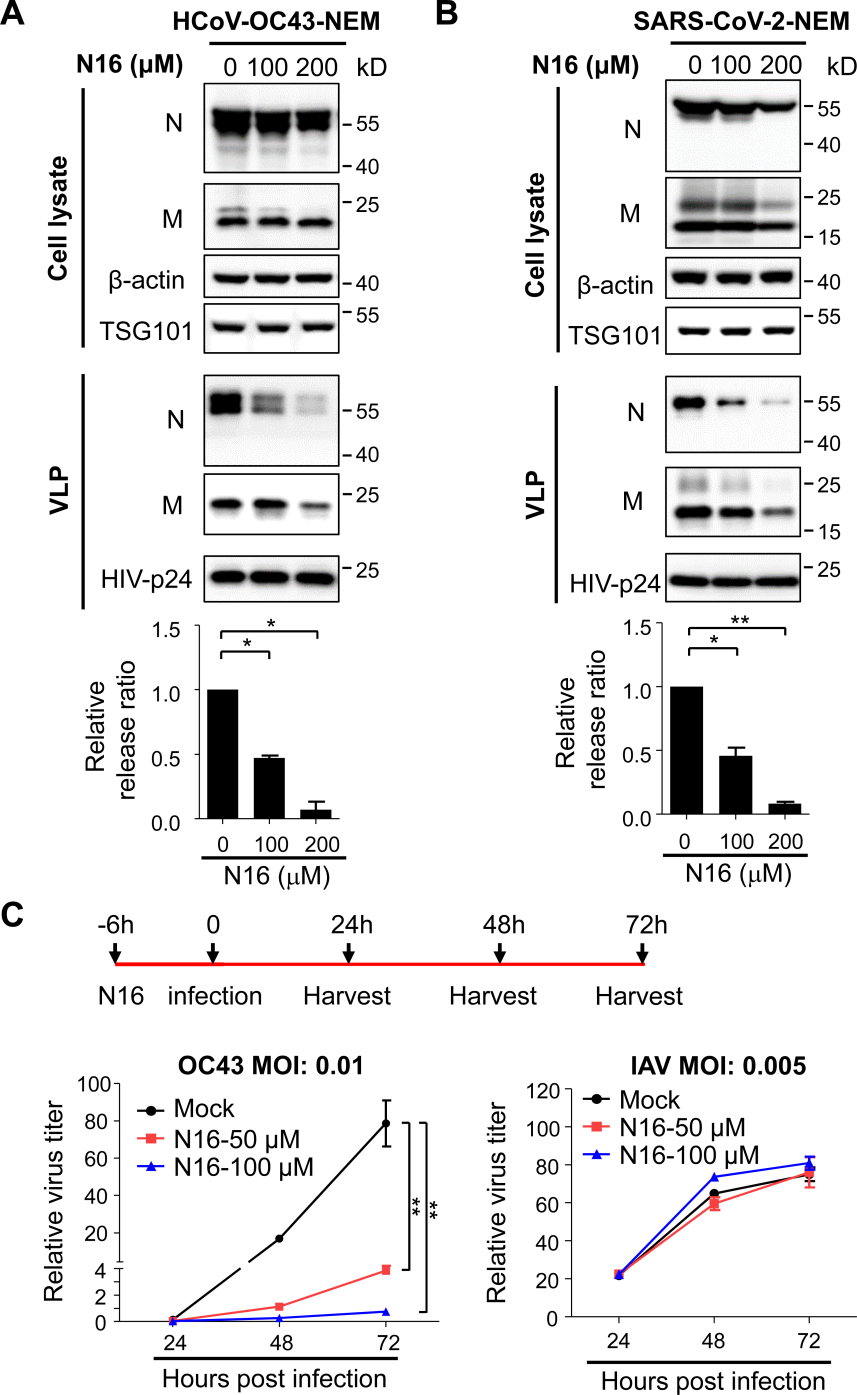
Tenatoprazole inhibits coronavirus production. (**A** and **B**) Tenatoprazole (N16) inhibits the VLP production of HCoV-OC43 (**A**) and SARS-CoV-2 (**B**). 293T cells were treated with N16 for 6 h and then transfected with the pNEM plasmid. At 48 h post-transfection, culture supernatants were collected. HIV-1 pseudovirus particles were added to serve as a control for sample handling. The relative VLP release ratio was calculated as described in the legend to Fig. S2. The relative release ratio from control cells was set as 1. Data presented are means ± SEM of two independent experiments.**C**) N16 inhibits the replication of HCoV-OC43. 293T cells were treated with N16 for 6 h and then infected with the virus indicated. At the time points indicated, relative virus titers in the culture supernatants were measured. Data presented are representative of three independent experiments.* denotes p < 0.05 and ** denotes p < 0.01.

The foregoing results showed that TSG101 knockdown impaired coronavirus VLP production and HCoV-OC43 replication. We reasoned that a TSG101 antagonist might inhibit the production of coronaviruses and thus viral replication. Tenatoprazole (N16), a TSG101 antagonist, has been reported to inhibit the replication of a variety of viruses that employ the ESCRT pathway for virus production, such as HIV-1 and EBV (50, 51). We first explored whether N16 could inhibit the VLP production of HCoV-OC43 and SARS-CoV-2. 293T cells were pretreated with N16 and then transfected with the pNEM construct and cultured in the presence of N16. The culture supernatant was collected. HIV-1 VLP, which was produced separately in 293T cells, was added to the coronavirus VLP-containing culture supernatant to serve as a control for sample handling. The VLPs in the culture supernatant were purified through ultracentrifugation, and viral protein levels in the VLPs and producer cells were measured. N16 modestly decreased the levels of N or M in the producer cells and significantly reduced the amount of the released VLP for both HCoV-OC43 (Fig. 5A) and SARS-CoV-2 (Fig. 5B) in a dose-dependent manner. These results indicated that the TSG101 antagonist N16 inhibits β-coronavirus VLP production.

To explore whether N16 inhibits the spreading of replication-competent β-coronavirus, 293T cells were infected with HCoV-OC43 in the presence of N16. In a control experiment, N16-treated cells were infected with influenza A virus. Virus titers in culture supernatants were monitored. Indeed, N16 significantly inhibited the spreading of HCoV-OC43 (Fig. 5C). In comparison, no obvious inhibitory effect of N16 against influenza virus was observed (Fig. 5C), demonstrating that the inhibitory effect against HCoV-OC43 was not caused by general cytotoxicity. These results indicated that TSG101 antagonist N16 inhibits HCoV-OC43 replication.

## DISCUSSION

β-Coronaviruses rely on host factors for replication (52–54). While the host factors involved in the entry process have been extensively studied, the host factors involved in virion assembly and egress are largely unknown. In this report, we showed that ESCRT is required for β-coronavirus virion assembly and egress. Knockdown of TSG101, VPS28, MVB12A, CHMP6, or VPS4A significantly inhibited VLP production (Fig. S2 to S6) and virus replication (Fig. 1; Fig. S7 and S8). Furthermore, knockdown of these ESCRT factors resulted in the disruption of the virion assembly and egress at different stages (Fig. 2), indicating that ESCRT is involved in multiple steps of coronavirus virion production.

Coronavirus N interacted with ESCRT-I component TSG101 (Fig. 3; Fig. S10). TSG101 knockdown significantly impaired coronavirus VLP production (Fig. S2A, S3A, S4A, S5A, and S6A) and HCoV-OC43 replication (Fig. 1A; Fig. S7 and S8). TEM analysis revealed that TSG101 depletion resulted in immature particles that remained connected to the membrane via stalks, indicating a disruption in the assembly process (Fig. 2A and C), reminiscent of the effect of TSG101 depletion on HIV-1 virion formation (47, 55, 56). It is well established that viruses can recruit TSG101 through the P[T/S] AP motif, and increasing lines of evidence indicate that viruses can recruit TSG101 through other motifs (41, 57–60). In addition, it is well established that TSG101 has ubiquitin-binding motifs that can facilitate the recognition and recruitment of ubiquitinated cargo (61) and that ubiquitin can functionally compensate for the absence of a viral late domain (62). It has been reported that coronavirus N can be ubiquitinated (63, 64). It is possible that coronavirus N could also recruit TSG101 through ubiquitination. How TSG101 dictates coronavirus virion assembly and how N recruits TSG101 await further investigation.

Coronavirus M interacted with the ESCRT-I component VPS28 (Fig. 4; Fig. S11). VPS28 knockdown impaired coronavirus VLP production (Fig. S2A, S3A, S4A, S5A, and S6A) and HCoV-OC43 replication (Fig. 1B; Fig. S7E, F and S8B). TEM analysis revealed that in VPS28 knockdown cells, virion assembly was nearly but not fully completed (Fig. 2B and C). How VPS28 is involved in virion assembly is not clear yet. It has been reported that fusing VPS28 to EIAV Gag could functionally replace the late YPX(n)L motif of the virus (65–69), suggesting that VPS28 could mediate the interaction between ESCRT and certain viruses. As an ESCRT-I component, VPS28 interacts with TSG101 and VPS37 (21, 70–73), as well as the ESCRT-III component CHMP6 (74, 75). Interaction of coronavirus M with VPS28 can be expected to recruit ESCRT to the virus assembly sites. However, how coronavirus M interacts with VPS28 and how VPS28 participates in virion production await further investigation.

Downregulation of MVB12A and CHMP6 impaired coronavirus VLP production (Fig. S2 to S6) and HCoV-OC43 replication (Fig. 1; Fig. S7, S8C and D). It has been proposed that human MVB12 is an important but not absolutely essential subunit of human ESCRT-I (56). Loss of MVB12 resulted in a partial defect in MVB sorting and mistargeting of ESCRT-I to the vacuolar lumen (76). In this report, MVB12A depletion seemed to negatively affect the formation of cellular vesicles and thus the egress of virion particles. CHMP6, a component of ESCRT-III, has been reported to regulate cargo sorting and participate in MVB formation (77). We speculate that CHMP6 depletion resulted in abnormal vesicle formation and thus inhibited coronavirus virion egress. Further investigation is needed to understand how ESCRT is involved in the coronavirus virion egress process.

In summary, we showed here that β-coronaviruses recruit ESCRT for virion assembly and release. The observation that TSG101 antagonist tenatoprazole inhibited the VLP production of β-coronaviruses and the replication of HCoV-OC43 (Fig. 5) suggests that the interaction interface between coronaviruses and ESCRT could be a target for the development of broad-spectrum anti-coronavirus therapeutics.

## MATERIALS AND METHODS

### Cell culture

293T (ATCC CRL-11268) and A549 (ATCC CCL-185) cells were maintained in Dulbecco’s modified Eagle’s medium (DMEM) (Invitrogen) supplemented with 10% fetal bovine serum (FBS) (Gibco) at 37°C, 5% CO_2_. In HCoV-OC43 infection experiments, 293T and A549 cells were maintained in DMEM supplemented with 2% FBS at 33°C, 5% CO_2_, during viral infection.

### siRNAs and siRNA transfection

All the siRNA sequences are listed in Table S3. The control siRNA and gene-specific siRNAs were obtained from JTSBIO Co., Ltd (Wuhan, China). The siRNAs were transfected into cells using Lipofectamine 2000 (Thermo Fisher) at a concentration of 25 nM following the manufacturer’s instruction. The siRNAs targeting TSG101, CHMP1A/B, CHMP2A/B, CHMP3, CHMP4A/B, CHMP5, CHMP6, CHMP7, and ALIX were initially reported by Morita et al. (78). The siRNAs targeting VPS37A/C were initially reported by Stefani et al. (79). The siRNA targeting VPS4A was initially reported by Kieffer et al. (80). The siRNAs targeting VPS28, MVB12A/B, and VPS37B were designed with BLOCK-iT RNAi Designer (Thermo Fisher).

To test the effect of ESCRT component knockdown on VLP production, 293T cells were seeded in 60 mm tissue culture dishes (2 × 10^6^ cells/per dish), cultured for 16 h, transfected with siRNA for 4 h, passaged to 60 mm tissue culture dishes (2 × 10^6^ cells/per dish) at 10 h post-transfection, and cultured for 24 h. The cells were then transfected with the same siRNA together with the pNEM construct. At 48 h post-transfection, cells were lysed, and cell lysates were subjected to Western blotting. The culture supernatants were collected, and HIV-1 VLP was added to serve as a control for sample handling. The samples were concentrated through 20% sucrose ultracentrifugation and analyzed by Western blotting. The relative protein levels were determined using ImageJ (National Institutes of Health) software. The VLP release ratio was calculated as the N protein level in the culture supernatant divided by that in the cell lysate. The relative release ratio of the VLP produced from control cells was set as 1.

To test the effects of ESCRT component knockdown on viral replication, 293T cells were transfected twice with siRNA with or without a rescue expression construct, and A549 cells were transfected once with siRNA with or without a rescue expression construct. The cells were then infected with HCoV-OC43 or IAV-Gluc. Culture supernatants were collected to measure relative virus titers at various time points. The cells were collected at 72 h post-infection for Western blot analysis.

### Antibodies

Antibodies used in this study are listed in Table S4.

### Virus infection and titration

293T or A549 cells were infected with HCoV-OC43 (strain VR1558) (81) for 2 h in DMEM supplemented with 2% FBS at 33°C. To measure the relative virus titer, the viral RNA in the culture supernatant of infected cells was extracted with AFTSpin Viral DNA/RNA Extraction Kit (Abclonal, cat. no. RK30113), followed by quantification with the one-step Quantitative TaqMan PCR (one-step qPCR) assay. The reaction was performed on Applied Biosystems QuantStudio 6 Real-Time PCR System (ABI) with forward primer OC43-M-F: 5′-ATGTTAGGCCGATAATTGAGGACTAT-3′, reverse primer OC43-M-R: 5′-AATGTAAAGATGGCCGCGTATT-3′, and OC43-M-probe: 5′-FAM-CATACTCTGACGGTCACAAT-TRMRA-3′ (initially reported by Vijgen and colleagues [82]), under the following conditions: UDG reaction at 25°C for 5 min, reverse transcription reaction 50°C for 5 min, denature at 95°C for 3 min, and 45 cycles of amplification (15 s at 95°C and 34 s at 60°C). The qRT-PCR reaction was carried out in a 20 µL reaction mixture with 3 µL extracted RNA, 12.4 µL of ABScript III One Step RT-qPCR Probe Kit with UDG V5 (Abclonal, cat. no. RK20412). The reporter dye (FAM) signal was measured against the internal reference dye (ROX) signal to normalize the signals for non-PCR-related fluorescence fluctuations that occur from well to well.

Cell lysate RNA was extracted using TRIzol reagent by following the manufacturer’s instructions and reverse transcribed with Moloney murine leukemia virus (MLV) reverse transcriptase using random primers. The RNA levels were measured by SYBR green real-time PCR in the Applied Biosystems QuantStudio 6 Real-Time PCR System (ABI) using the following program: 95°C for 5  min, one cycle; 95°C for 15 s, 60°C for 20 s, and 72°C for 20 s, 40 cycles; and 95°C for 15 s, 60°C for 1 min, 95°C for 15 s, one cycle. Sequences of the PCR primers are as follows: OC43-M-F: 5′-ATGTTAGGCCGATAATTGAGGACTAT-3′, OC43-M-R: 5′-AATGTAAAGATGGCCGCGTATT-3′; β-actin-F: 5′-GGAAATCGTGCGTGACATTAA-3′, β-actin-R: 5′-AGGAAGGAAGGCTGGAAGAG-3′. Intracellular viral M RNA level was measured against the internal reference β-actin to normalize. The relative viral RNA levels from control cells were set as 1.

The production and titration of IAV-Gluc have been previously described (83). 293T cells were infected with IAV-Gluc for 2 h in DMEM supplemented with 2% FBS at 33°C. To monitor IAV-Gluc replication, aliquots of the culture supernatant were taken at various time points to measure Gaussia luciferase activity.

### TEM

Virus particles or VLPs in a 3 µL sample solution were deposited onto a copper mesh (mesh size of 230) with a carbon-coated grid which had been treated with a glow discharger. After incubation for 60 s, the residual sample solution was blotted off with a filter paper from the grid edge. Uranyl acetate (2%) solution was added onto the grid quickly, and the stain was then blotted away quickly with filter paper. After repeating this process for another time, the grid was stained with 3 µL 2% uranyl acetate solution for 60 s, after which the stain solution was removed with filter paper. The grid was dried in air and observed using a transmission electron microscope (Thermo Fisher Tecnai Spirit, 120kV) equipped with an EMSIS Veleta camera (2K × 2K).

To observe the morphology of virus particles in cells, 293T cells seeded in a 60 mm culture dish were transfected with siRNA twice as described above and infected with HCoV-OC43 (multiplicity of infection [MOI] = 0.1) for 2 h. At 48 h post-infection, cells were collected, washed with phosphate-buffered saline (PBS), and centrifuged at 2,000 × *g* at 4°C for 2 min. The procedures for further sample processing and TEM analysis have been reported previously (84). Briefly, cells were fixed with 2.5% (vol/vol) glutaraldehyde in phosphate buffer (0.1 M, pH 7.4) twice and then in ddH_2_O twice at 4°C. The cells were post-fixed with 1% (wt/vol) OsO_4_ and 1.5% (wt/vol) potassium ferricyanide aqueous solution for 2 h at 4°C, dehydrated through a series of ethanol solution (30%, 50%, 70%, 80%, 90%, and 100% × 2, 6 min in each solution) into pure acetone (2 × 6 min). Samples were infiltrated in graded mixture (3:1, 1:1, and 1:3) of acetone and SPI-PON812 resin (21 mL SPI-PON812, 12 mL Dodecenyl Succinic Anhydride and 11 mL Nadic Methyl Anhydride), then pure resin. The cells were embedded in pure resin with 1.5% N, N Dimethylbenzylamine and polymerized for 12 h at 45°C and then 48 h at 60°C. The ultrathin sections (70 nm) were sectioned with a microtome (Leica EM UC6), double-stained by uranyl acetate and lead citrate, and examined by a transmission electron microscope (FEI Tecnai Spirit 120 kV).

### Co-immunoprecipitation assay

293T cells were transfected with protein expression constructs using Xpregen following the manufacturer′s instructions (Beijing Yu-Feng Biotechnology, cat. no. ND01). At 48 h post-transfection, cells were lysed in PBS (Gibco) containing 0.03% n-dodecyl-b-D-maltoside (DDM; Anatrace, cat no. D310LA) and protease inhibitor cocktail (Roche, cat. no. 11873–580-001). The lysate was clarified and mixed with the antibody and protein G beads (GE Healthcare, cat. no. 17–0618-05) or anti-Flag (Sigma-Aldrich, cat. no. A2220)/anti-myc (Sigma-Aldrich, cat. no. A7470) affinity gel at 4°C for 3 h. The beads were washed with PBS containing 0.03% DDM five times, and the bound proteins were resolved on SDS-PAGE electrophoresis, transferred to polyvinylidene difluoride (PVDF) membrane, and detected by Western blotting.

### Protein purification and pull-down assay

SARS-CoV-2 N was bacterially expressed in BL21 (DE3) Chemically Competent Cell (Transgene Biotech, cat. no. CD601-01) with a 6× His-tag fused to the N-terminus. Coronavirus M proteins were bacterially expressed with a 6× His-tag and a Bril-tag fused to the N-terminus. The proteins were partially purified through a Ni (Sigma-Aldrich, cat. no. GE17-5318-01) column. The elution buffer for the N protein was 50 mM Tris-HCl, pH 7.5, 500  mM NaCl, 500 mM imidazole, and 5% glycerol. The procedure of M protein purification was the same, except that 0.03% DDM was added to the lysis and elution buffers.

Purified SARS-CoV-2 N or control recombinant protein was mixed with the lysate of cells transiently expressing Flag-TSG101 in PBS containing 0.03% DDM, incubated with anti-His antibody and protein G beads at 4°C for 3 h. The beads were washed with PBS containing 0.03% DDM five times, and the proteins were resolved on SDS-PAGE electrophoresis, transferred to the PVDF membrane, and detected by Western blotting. The procedure to analyze the ability of the M protein to pull down Flag-VPS28 was similar, except that M was immobilized with anti-Bril antibody.

### Confocal microscopy

To observe the cellular localizations of M and VPS28, plasmids expressing Flag-tagged M (SARS-CoV-2 M was not tagged) were transfected into 293T cells. At 24 h post-transfection, cells were fixed with 4% paraformaldehyde, washed with PBS, and permeabilized with 0.2% Triton X-100. The cells were then stained and photographed using a laser confocal microscope. VPS28 was stained with rabbit anti-VPS28 antibody and ABFlo 488-conjugated anti-rabbit secondary antibody. TSG101 was stained with CoraLite 594-conjugated TSG101 Monoclonal antibody. SARS-CoV-2 M was stained with mouse anti-M antibody and Alexa Fluor TRITC-conjugated anti-mouse secondary antibody, SARS-CoV-2 N was stained with mouse anti-N antibody and Alexa Fluor TRITC-conjugated anti-mouse secondary antibody, and Flag-tagged M was stained with mouse anti-Flag antibody and Alexa Fluor TRITC-conjugated anti-mouse secondary antibody. The cell nucleus was stained with 4′,6-diamidino-2-phenylindole (Beyotime Biotechnology; cat. no. C-1005).

To detect the cellular localization of VPS28/TSG101 upon HCoV-OV43 infection, 293T cells were mock infected or infected with HCoV-OC43 (MOI = 0.01). At 24 h post-infection, cells were fixed with 4% paraformaldehyde, washed with PBS, and permeabilized with 0.2% Triton X-100. The cells were then stained and photographed using a laser confocal microscope (Zeiss LSM980). VPS28 was stained with mouse anti-VPS28 antibody and Alexa Fluor TRITC-conjugated anti-mouse secondary antibody. TSG101 was stained with CoraLite 594-conjugated TSG101 monoclonal antibody. HCoV-OC43 N was stained with rabbit anti-N antibody and ABFlo 488-conjugated anti-rabbit secondary antibody.

### Quantification and statistical analysis

The band intensities were measured with ImageJ software. Unless otherwise indicated, the relative VLP release ratio was calculated as the relative N level in the culture supernatant divided by that in the cell lysate. The release ratio of the VLP from the control cells was set as 1. The data were analyzed with GraphPad Prism version 5 (GraphPad software). The error bars represent the mean ± SEM. *P* values were calculated using unpaired Student’s *t*-test. *, *P*  <  0.05; **, *P*  <  0.01; n.s. (non-significance), *P*  > 0.05.

## SUPPLEMENTAL MATERIAL

Data Set S1 mbio.00979-25-s0001.xlsx The raw data corresponding to Table S1.

Supplemental Material mbio.00979-25-s0002.pdf Supplemental methods, figures, and tables and caption for Data Set S1.

ASM does not own the copyrights to Supplemental Material that may be linked to, or accessed through, an article. The authors have granted ASM a non-exclusive, world-wide license to publish the Supplemental Material files. Please contact the corresponding author directly for reuse.
