# Supplementary material for: β-Coronaviruses exploit ESCRT for virion assembly and egress
Source: mBio. 2025 May 23;16(6):e00979-25. doi: 10.1128/mbio.00979-25 (PMC12153360; doi:10.1128/mbio.00979-25)
Supplement: Supplemental Material — Supplemental methods, figures, and tables and caption for Data Set S1. [file mbio.00979-25-s0002.pdf]

Supplemental materials for

β-coronaviruses exploit ESCRT for virion assembly and egress.

Yuanyuan Zhang[1,2], Linlong Huang[1], Chaoqi Ren[1], Weiyang Wang[1], Xinlu Wang[1*] and Guangxia Gao[1,2*]

E-mail: Guangxia Gao: gaogx@moon.ibp.ac.cn;

Xinlu Wang: wang_xl1978@aliyun.com

This PDF file includes:

Supplemental Methods
Figures S1 to S12
Tables S1 to S4

Other supporting materials for this manuscript include the following:

Data set S1

Supplemental Methods

Plasmid construction

In the VLP production system, the cDNAs encoding human coronavirus OC43 (HCoV-OC43, strain: ATCC VR-759, serotype: OC43) viral protein N (nucleocapsid protein, NC_006213.1: 29079-30425), M (membrane protein, NC_006213.1: 28377-29069) and E (envelope protein, NC_006213.1: 28108-28362), MERS-CoV (strain: HCoV-EMC, isolate: HCoV-EMC/2012) viral protein N (NC_019843.3: 28566-29807), M (NC_019843.3: 27853-28512) and E (NC_019843.3: 27590-27838), human coronavirus HKU1 (isolate: HKU1) viral protein N (NC_006577.2: 28320-29645), M (NC_006577.2: 27633-28304) and E (NC_006577.2: 27373-27621) were purchased from Tsingke Biotechnology Co., Ltd (Beijing). The coding sequences of SARS-CoV-2 (isolate: Wuhan-Hu-1) viral protein N (NC_045512.2: 28274-29533), M (NC_045512.2: 26523-27191) and E (NC_045512.2: 26245-26472) were PCR-amplified from a cDNA library of SARS-CoV-2 (kindly provided by Pro Hong Zhang, Institute of Biophysics). The coding sequences of N, M and E were cloned into a eukaryotic expression vector pCMV to construct a plasmid expressing N, M and E (pNEM). In this construct, the coding sequences of N and M are under the transcriptional control of CMV and EF1α promoters, respectively. The coding sequence of E is downstream of that of N with its translation initiated by EMCV IRES. A Flag-tag was fused to the N-terminus of the M protein of OC43 and HKU1.

To express proteins for the coimmunoprecipitation assays, the coding sequences of the ESCRT-I components TSG101(NM_006292.4), VPS28 (NM_016208.4), MVB12A (NM_138401.4), MVB12B (NM_033446.3), VPS37A (NM_152415.3), VPS37B (NM_024667.3) and VPS37C (NM_017966.5) were PCR-amplified from a cDNA library of 293T cells and cloned into pCMV with a Flag-tag at the N-terminus. The coding sequence of N was cloned into pCMV. The coding sequence of M was cloned into pCMV with a myc-tag at the N-terminus.

To confirm the specificity of the siRNA, a rescue expression construct that cannot be targeted by the siRNA was generated. The coding sequence targeted by the siRNA was synonymously mutated and cloned into expression vectors. The mutated sequences are listed below:

TSG101: 5'-CCTCCAGTCTTCTCTCGTC-3';
MVB12A: 5'-AGAAACGCATGTGTGTGAAGCTGTT-3';
VPS28: 5'-AGCCGGAGCTGTATGAGGAAGTGAA-3';
CHMP6: 5'-GGAAATGAGTGTCTGAACA-3';
VPS4A: 5'-CCGAGAAGCTGAAGGATTA-3'.

Virion like particles (VLP) purification for MS/TEM

Culture supernatant was collected at 48 h posttransfection, filtered through 0.45 mm Sterile Filter Unit (Millipore) and centrifuged through 20%

sucrose cushion with P32ST rotor (Hitachi himac CP 70MX) at 100000g for 2 h. The pellet was resuspended and subjected to 10%~50% sucrose continuous density gradient centrifugation at 20000g for 16 h. Eleven fractions were collected, 1 ml each. The distribution of viral proteins in these fractions was analyzed by Western blotting. Fractions 7 and 8 of HCoV-OC43 VLP, and fractions 6 and 7 of SARS-CoV-2 VLP were diluted with PBS and filtered through 100 kDa Centrifugal Filter Units (Millipore) to remove sucrose. The purified VLPs were used for transmission electron microscope (TEM) or mass spectrometry (MS) analysis. HCoV-OC43 virions inactivated with β-propiolactone and the control cell culture supernatants without virus particles were purified through the same procedure as VLPs.

LC-MS/MS analysis and identification of proteins

All nanoLC-MS/MS experiments were performed on the Orbitrap Exploris 480 (Thermo Scientific) equipped with an Easy n-LC 1200 HPLC system (Thermo Scientific). The peptides were loaded onto a 100 μm id×2 cm fused silica trap column packed in-house with reversed phase silica (Reprosil-Pur C18 AQ, 5 μm, Dr. Maisch GmbH) and then separated on an a 75 μm id×25 cm C18 column packed with reversed phase silica (Reprosil-Pur C18 AQ, 1.9 μm, Dr. Maisch GmbH). The peptides bounded on the column were eluted with a 73-min linear gradient. Solvent A was 0.1% formic acid in water and solvent B consisted of 80% acetonitrile and 0.1% formic acid. The segmented gradient was 4-9% B, 3 min; 9-20% B, 22 min; 20-30% B, 20 min; 30-40% B, 15 min; 40-95% B, 3min; 95% B, 10min at a flow rate of 300 nl/min.

The MS analysis was performed with Orbitrap Exploris 480 mass spectrometer with the FAIMS Pro interface (Thermo Scientific). FAIMS separations were performed with two compensation voltage (-45 and -65). With the data-dependent acquisition mode, MS data were acquired at a high resolution 60,000 (m/z 200) across the mass range of 350–1500 m/z. The target value was 3.00E+06 with a maximum injection time of 22 ms. Data dependent mode was selected as cycle time mode which was set as 2 seconds. The precursor ions were selected from each MS full scan with isolation width of 1.6 m/z for fragmentation in the Ion Routing Multipole with normalized collision energy of 28%. Subsequently, MS/MS spectra were acquired at resolution 15,000 at m/z 200. The target value was 7.50E+04 with a maximum injection time of 22 ms. The dynamic exclusion time was 40s. For nano electrospray ion source setting, the spray voltage was 2.0 kV; no sheath gas flow; the heated capillary temperature was 320 °C.

The raw data from Orbitrap Exploris 480 were analyzed with Proteome Discovery version 2.4.1.15 using Sequest HT search engine for protein identification. The Uniprot human and HCoV-OC43/SARS-CoV-2 protein database (updated on 11/2022) were used for searching the data from samples. Some important searching parameters were set as following: trypsin was selected as enzyme and two missed cleavages were allowed for searching; the mass tolerance of precursor was set as 10 ppm and the product ions tolerance

was 0.02 Da.; the cysteine carbamidomethylation was specified as fixed modification; the methionine oxidation was chosen as variable modification. FDR analysis was performed with Percolator and FDR <1% was set for protein identification.

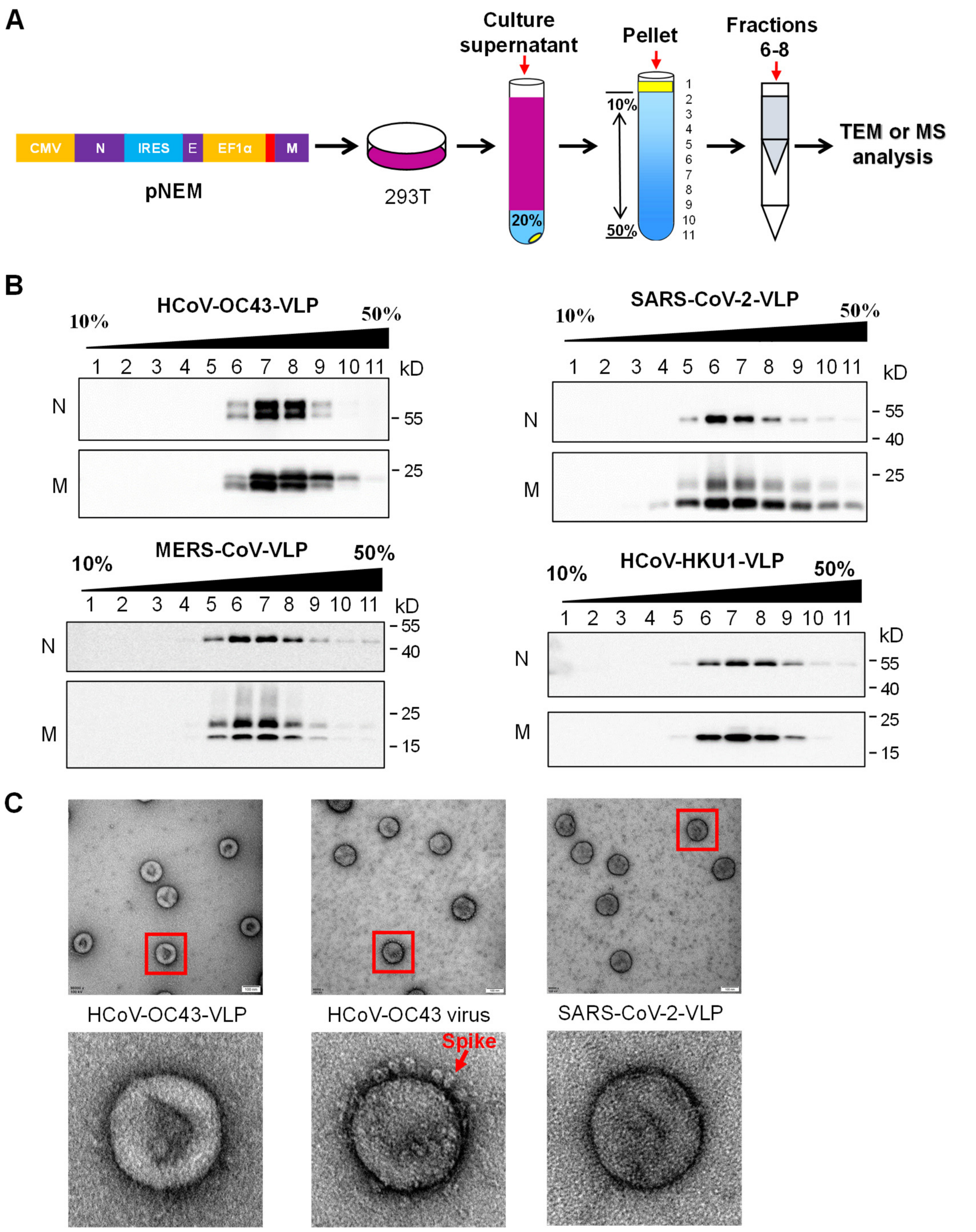

Fig. S1. Production of β-coronavirus virion like particles (VLP). (A) Schematic illustration of the procedure for production of β-coronavirus VLPs. The coding sequences of N, M and E were cloned into a eukaryotic expression vector pCMV to construct a plasmid expressing N, M and E (pNEM). In this construct, the coding sequences of N and M are under the transcriptional control of CMV and EF1α promoters, respectively. The coding sequence of E is downstream of N with its translation initiated by EMCV IRES. A Flag-tag was fused to the N-terminus of the M proteins of HCoV-OC43 and HCoV-HKU1 (the red square represents Flag-tag), and the M proteins of SARS-CoV-2 and MERS-CoV were not tagged. 293T cells were transfected with pNEM. At 48 h

posttransfection, culture supernatants were collected and purified through 20% sucrose cushions. The pellet was resuspended and subjected to 10%~50% sucrose velocity sedimentation centrifugation. Fractions containing VLPs were concentrated with 100 kD Centrifugal Filter Units. Purified VLPs were used for transmission electron microscopy (TEM) or mass spectrometry (MS) analysis. **(B)** The VLPs were subjected to 10%~50% sucrose velocity sedimentation centrifugation and fractions were collected. The viral protein levels in each fraction were analyzed by Western blotting. **(C)** TEM analysis of HCoV-OC43 VLP, HCoV-OC43 virion particles and SARS-CoV-2 VLP. HCoV-OC43 virions inactivated with β-propiolactone were purified through the same procedure as VLPs. The image on the lower panel shows the enlarged view of the selected area within a red rectangle. The arrow points to the HCoV-OC43 Spike. Scale bars: 100 nm.

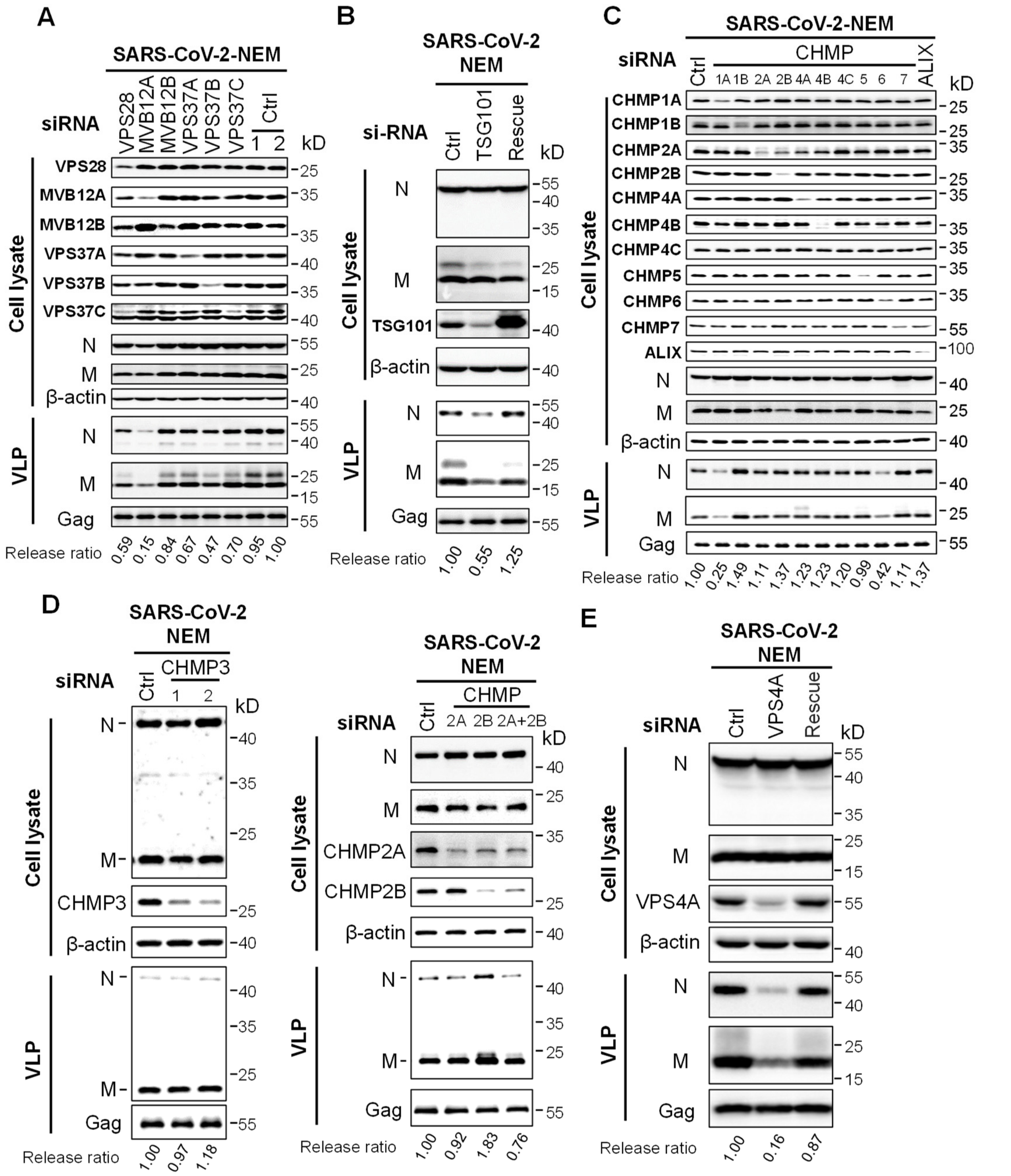

Fig. S2. Knockdown of certain ESCRT components impairs SARS-CoV-2 VLP production. 293T cells were first transfected with the siRNA indicated. The cells were then co-transfected with the same siRNA and pNEM. At 48 h posttransfection, cells were collected for Western analysis. Culture supernatants were collected. HIV-1 VLP, which was produced separately in 293T cells, was added to the culture supernatants to serve as a control for sample handling. The culture supernatants were purified through 20% sucrose cushion and analyzed for viral protein levels by Western blotting. The relative protein levels were measured using the ImageJ software. The relative VLP release ratio was calculated as the relative N level in culture supernatant divided by that in cell lysate. The release ratio of the VLP from control cells was set as 1. **(B)** 293T cells were transfected with a control siRNA or an siRNA

targeting TSG101 with or without a rescue TSG101-expressing construct that cannot be targeted by the siRNA. **(E)** 293T cells were transfected with a control siRNA or an siRNA targeting VPS4A with or without a rescue VPS4A-expressing construct that cannot be targeted by the siRNA. Data presented are representative of two independent experiments. Ctrl, control.

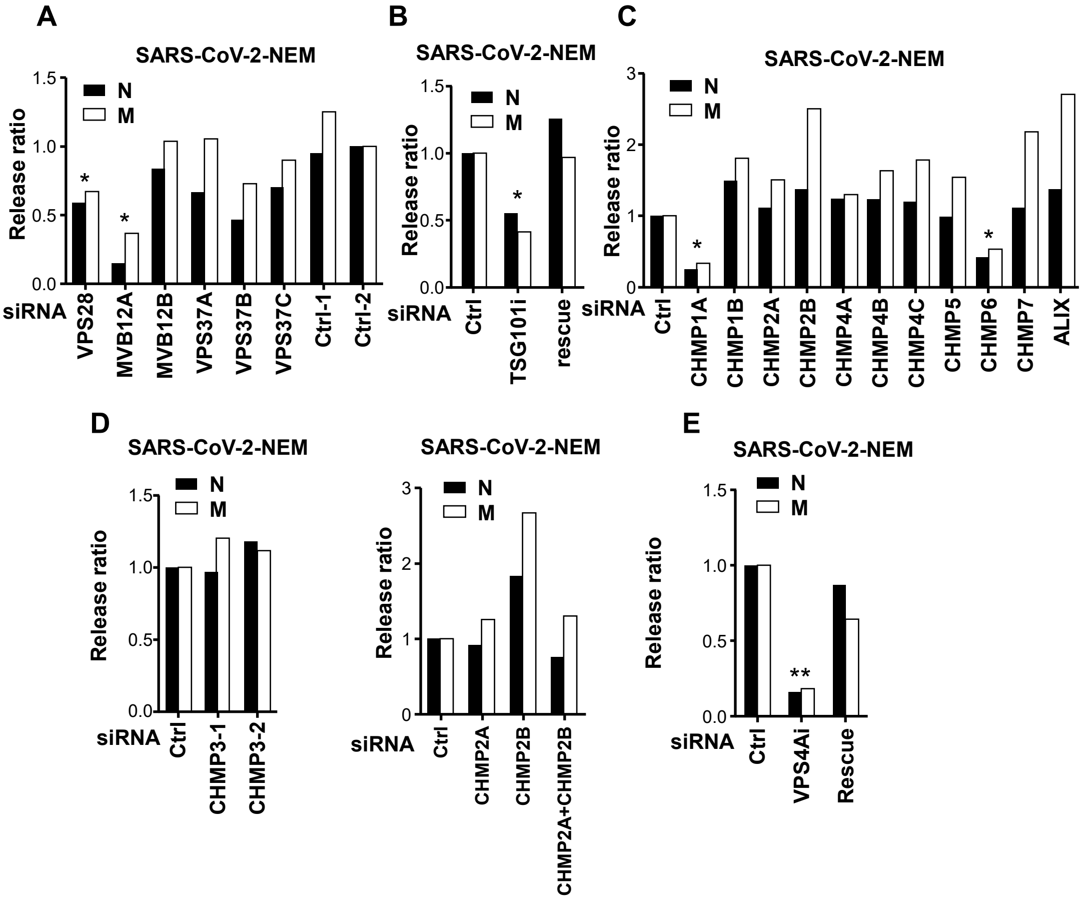

Fig. S3. Graphic presentation of the results in Figure S2. The relative VLP release ratios based on N levels and M levels in Fig. S2 were plotted. The relative release ratio of the VLP for the control cells was set as 1. * denotes $p < 0.05$ and ** denotes $p < 0.01$.

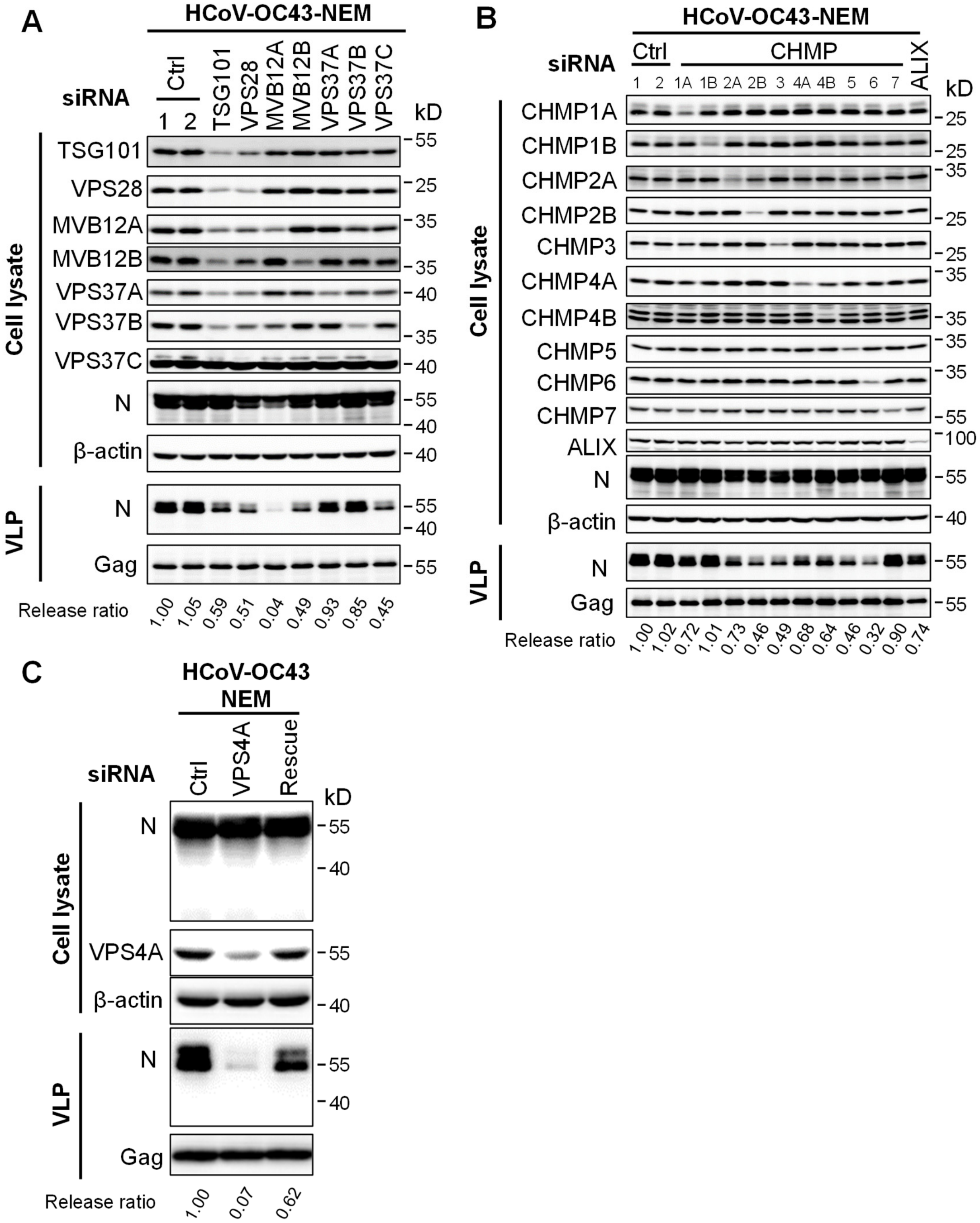

Fig. S4. Knockdown of certain ESCRT components impairs HCoV-OC43 VLP production. The relative VLP release ratio was calculated as described in the legend to Fig. S2. Data presented are representative of two independent experiments.

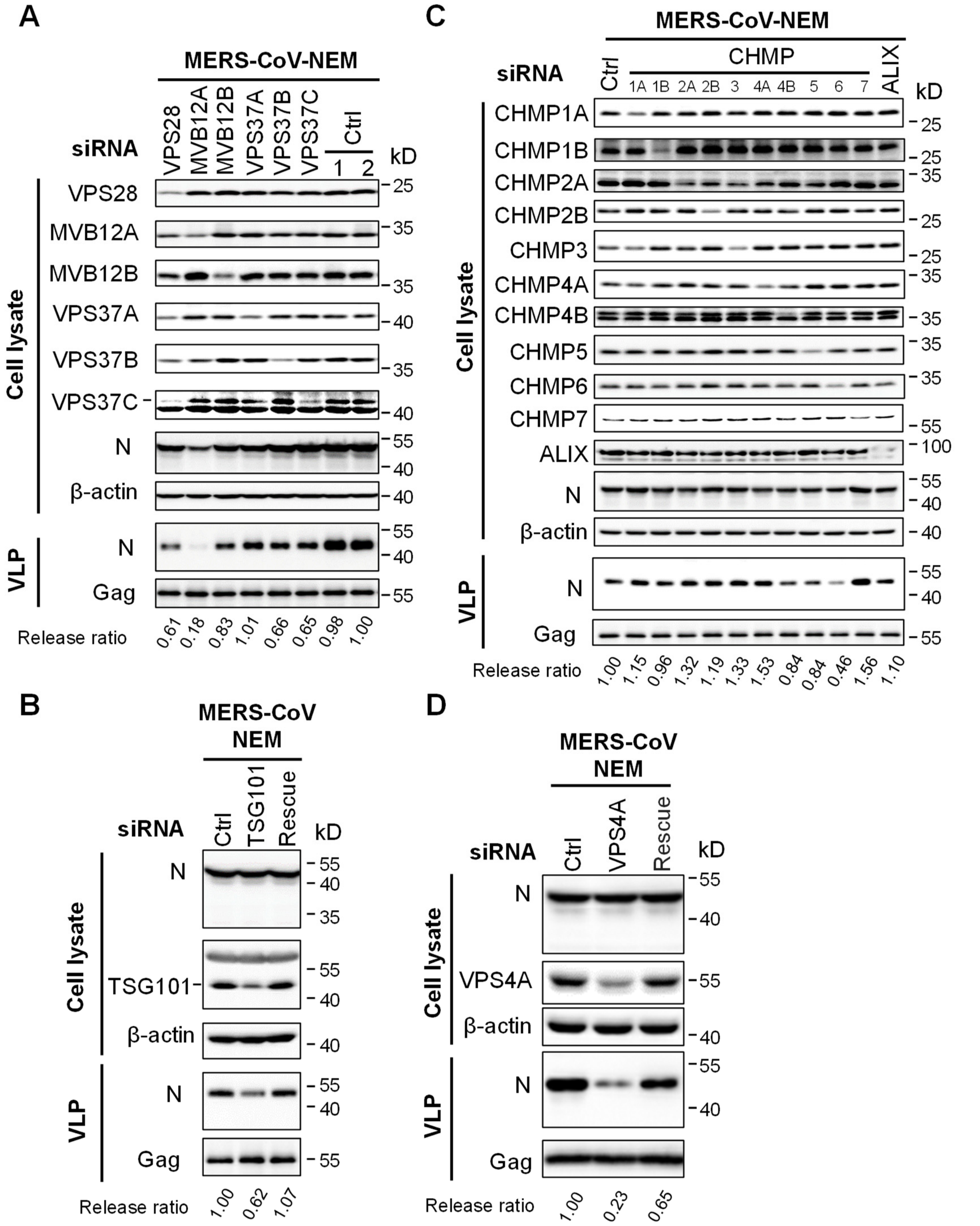

Fig. S5. Knockdown of certain ESCRT components impairs MERS-CoV VLP production. The relative VLP release ratio was calculated as described in the legend to Fig. S2. Data presented are representative of two independent experiments.

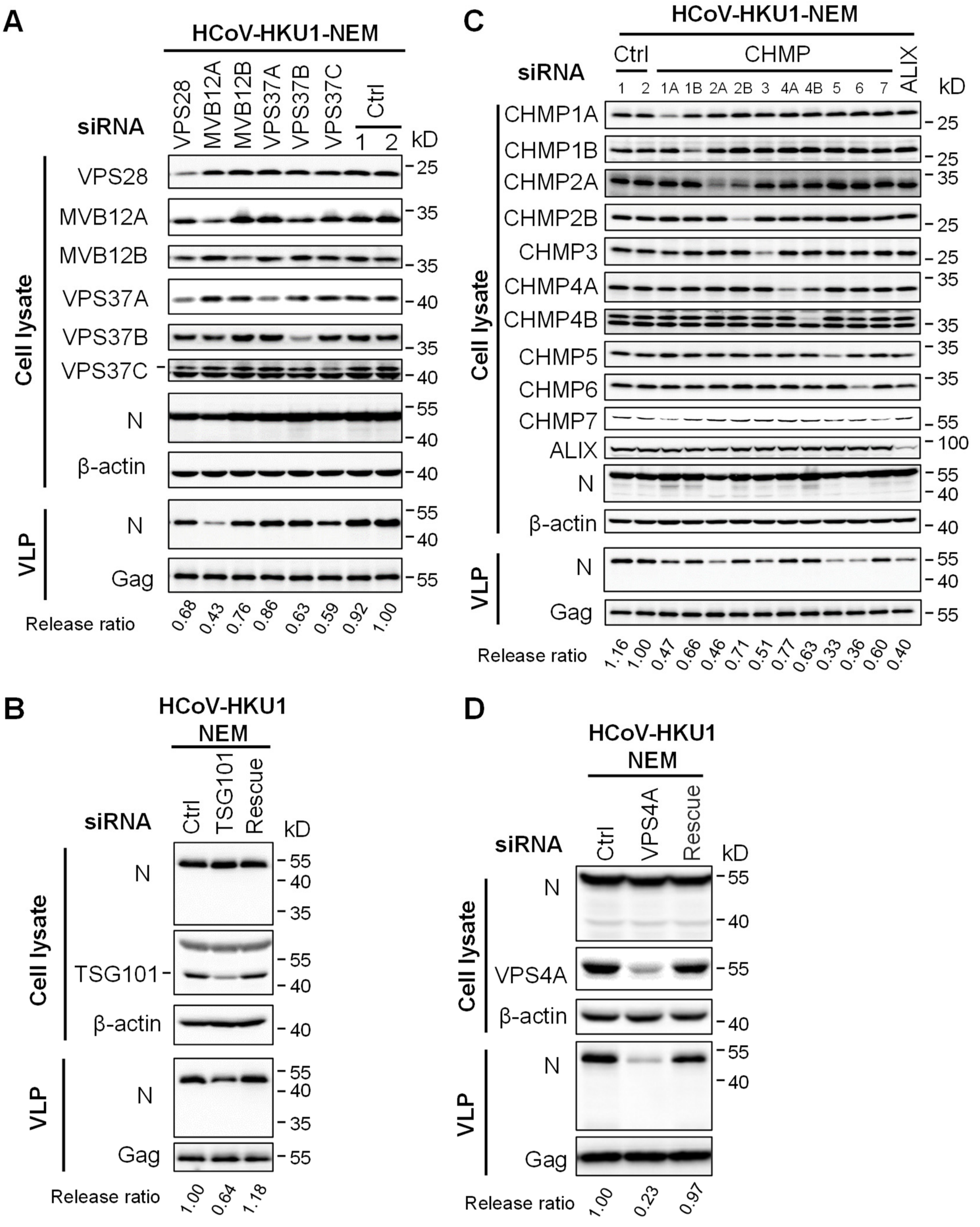

Fig. S6. Knockdown of certain ESCRT components impairs HCoV-HKU1 VLP production. The relative VLP release ratio was calculated as described in the legend to Fig. S2. Data presented are representative of two independent experiments.

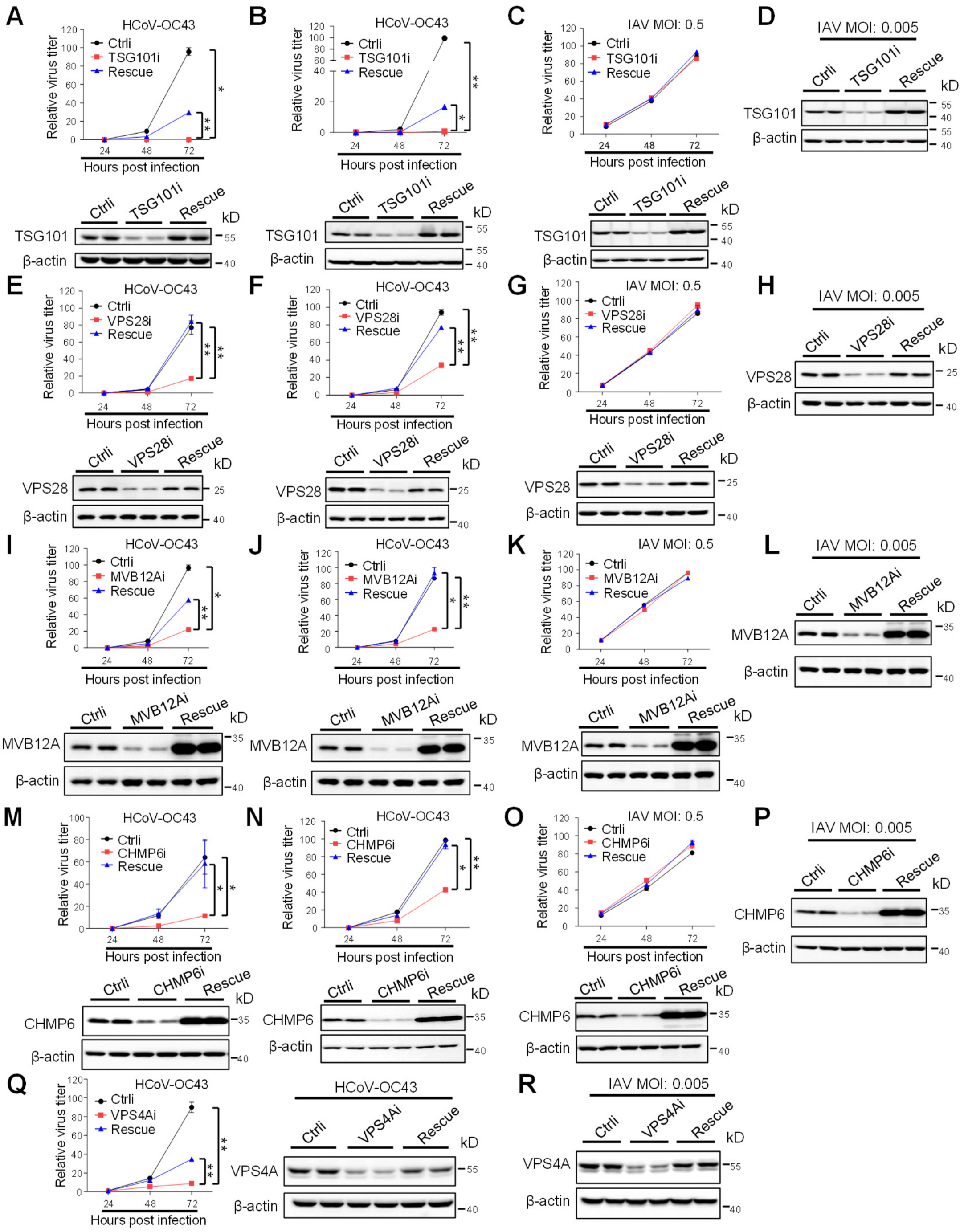

Fig. S7. Results of repeated experiments in Figure 1. 293T cells were transfected with the siRNA indicated with or without a rescue expression construct. The cells were then infected with HCoV-OC43 or IAV-Gluc. Culture supernatants were collected to measure relative virus titers at the time points indicated. The cells were collected at 72 h postinfection for Western analysis. MOI, multiplicity of infection; Ctrl, control; R, rescue. * denotes $p < 0.05$ and ** denotes $p < 0.01$.

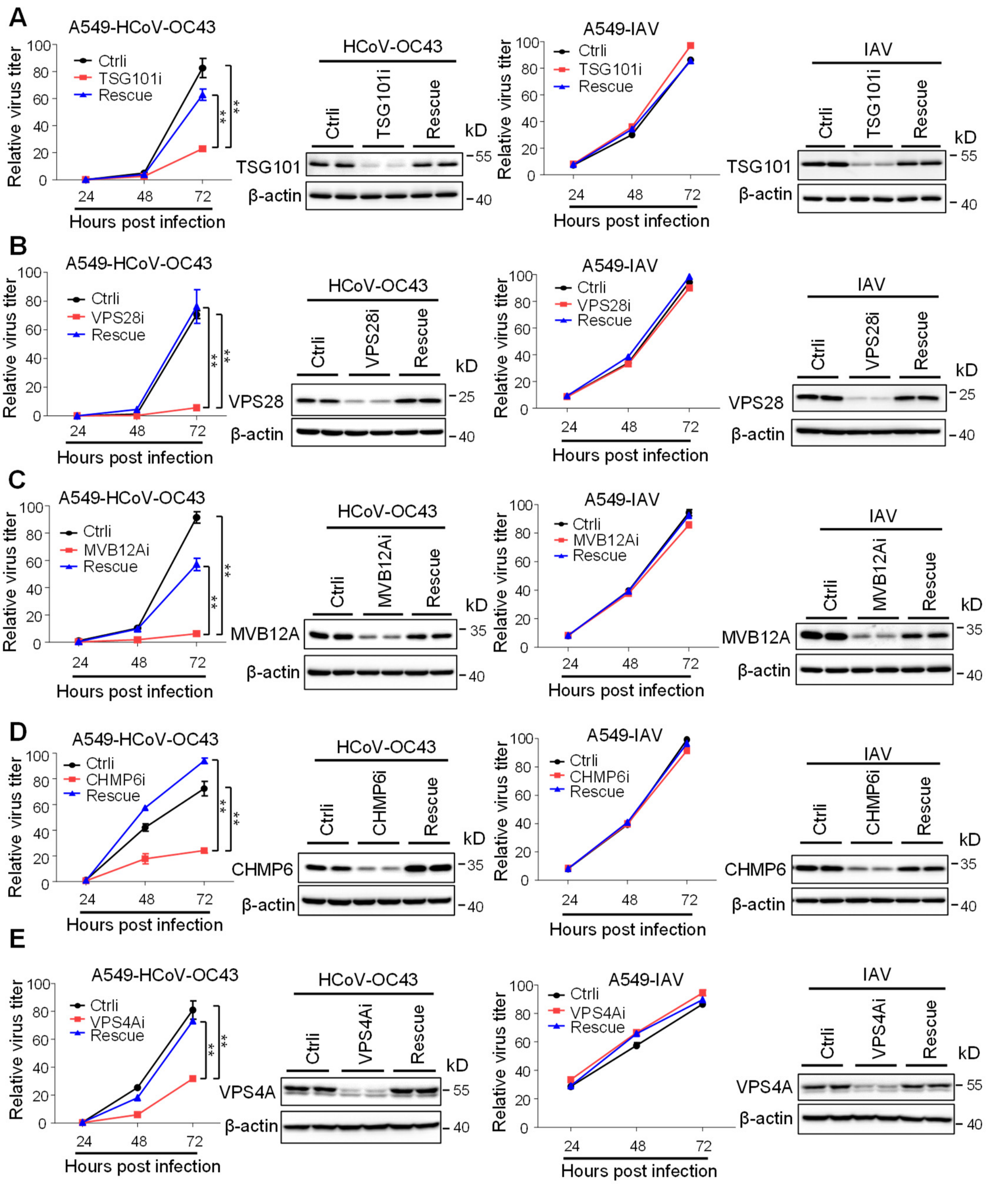

Fig. S8. Knockdown of certain ESCRT components impairs the replication of HCoV-OC43 in A549 cells. A549 cells were transfected with the siRNA indicated with or without a rescue expression construct. The cells were then infected with HCoV-OC43 or IAV-Gluc. Culture supernatants were collected to measure relative virus titers at the time points indicated. The cells were collected at 72 h postinfection for Western analysis. ** denotes p< 0.01. Data presented are representative of two independent experiments.

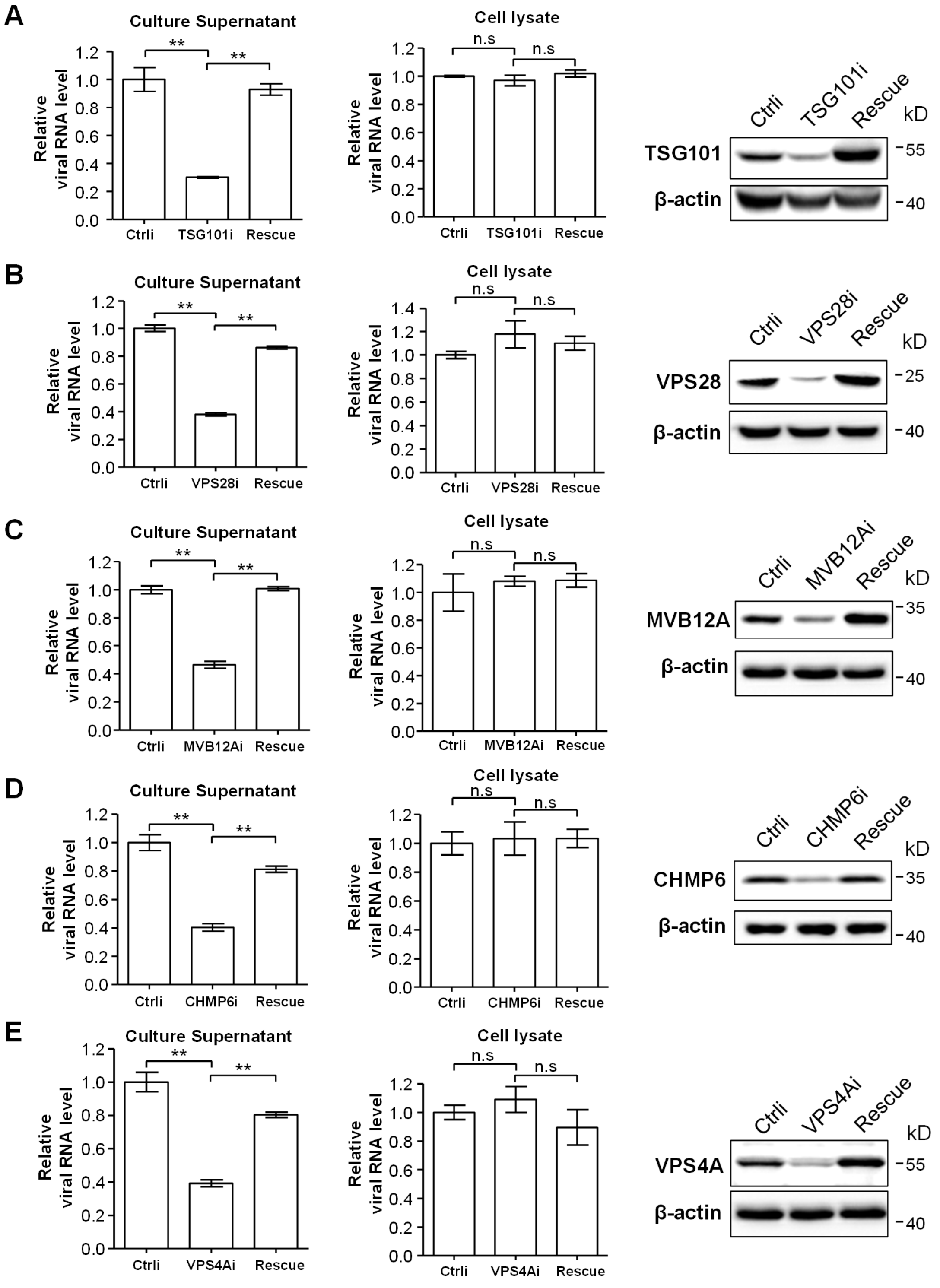

Fig. S9. Knockdown of certain ESCRT components impairs HCoV-OC43 release. 293T cells were transfected with the siRNA indicated with or without a rescue expression construct. The cells were then infected with HCoV-OC43 (MOI=4). At 24 h postinfection, the viral M RNA levels in the culture supernatants and cell lysates were measured. The cell lysates were analyzed by Western blotting. ** denotes $p < 0.01$ and n.s. denotes $p > 0.05$.

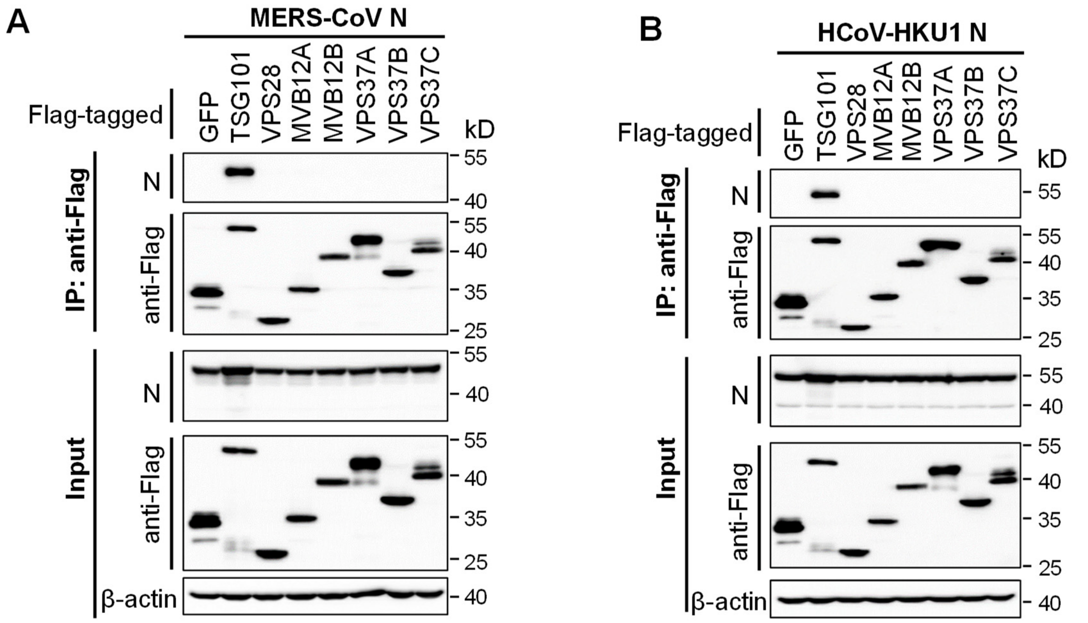

Fig. S10. TSG101 interacts with N of MERS-CoV and HCoV-HKU1. The interactions of the N proteins of MERS-CoV (A) and HCoV-HKU1 (B) with TSG101 were analyzed as described in the legend to Fig. 3A. The N proteins were detected with the corresponding rabbit polyclonal antibodies. Data presented are representative of two independent experiments.

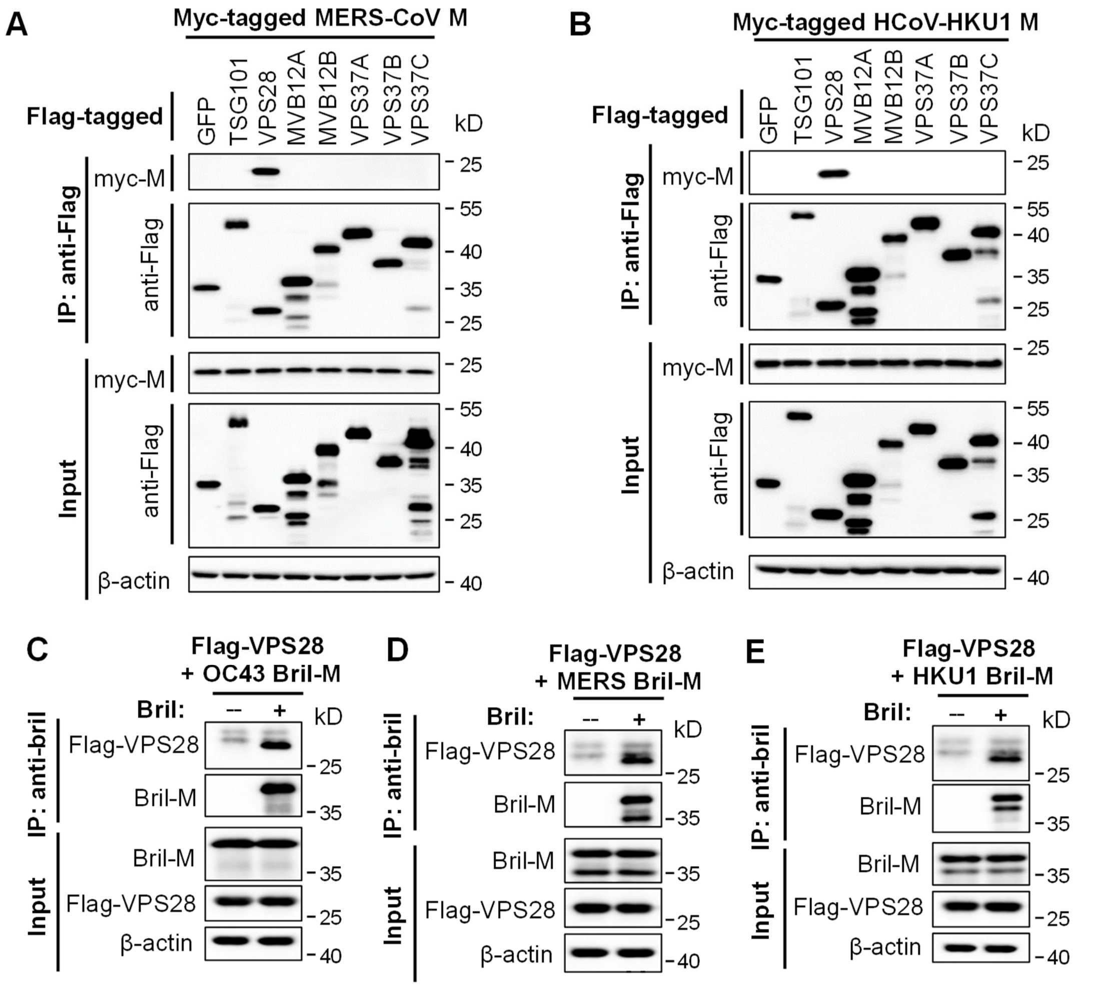

Fig. S11. VPS28 interacts with coronavirus M. (A-B) The interactions of the M proteins of MERS-CoV (A) and HCoV-HKU1 (B) with VPS28 were analyzed as described in the legend to Fig. 4A. **(C-E)** The interactions of Flag-VPS28 with the recombinant Bril-M proteins were analyzed as described in the legend to Fig. 4D. Data presented are representative of two independent experiments.

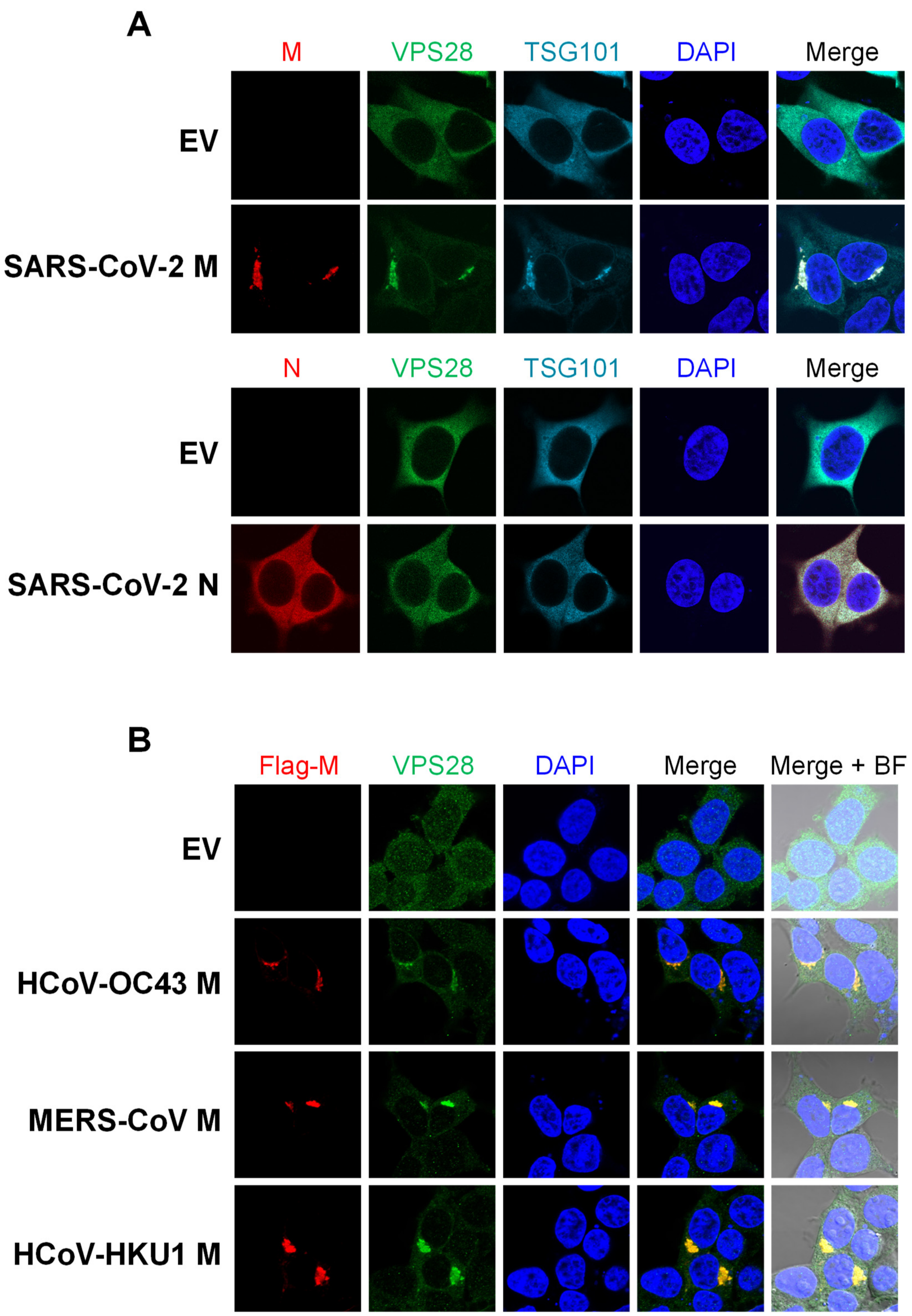

Fig. S12. VPS28 colocalized with coronavirus M. (A) SARS-CoV-2 M or N was transiently expressed in 293T cells. The cells were fixed in paraformaldehyde at 24 h posttransfection. VPS28 was stained with rabbit anti-VPS28 antibody and ABFlo 488-conjugated anti-rabbit secondary antibody (green). TSG101 was stained with CoraLite® 594-conjugated TSG101 Monoclonal antibody (original). SARS-CoV-2 M or N was stained with mouse anti-M or anti-N antibody and Alexa Fluor TRITC-conjugated anti-mouse

secondary antibody (red). Fluorescence images were obtained with confocal fluorescence microscopy (LSM980). **(B)** Endogenous VPS28 colocalized with coronavirus M. Flag-tagged M indicated was transiently expressed in 293T cells. The cells were fixed in paraformaldehyde and subjected to immunostaining analysis at 24 h posttransfection. Flag-tagged M was stained with mouse anti-Flag antibody and Alexa Fluor TRITC-conjugated anti-mouse secondary antibody (red). The endogenous VPS28 was stained with rabbit anti-VPS28 antibody and ABFlo 488-conjugated anti-rabbit secondary antibody (green). Fluorescence images were obtained with confocal fluorescence microscopy (LSM700). BF, bright field.

Table S1. TSG101 is detected in purified coronavirus VLP and virion samples. Purified HCoV-OC43 VLP, SARS-CoV-2 VLP and HCoV-OC43 virions were subjected to MS analysis. The culture supernatant of the control cells without virion particles was purified through the same procedure to serve as a control (Control sample). The information about viral proteins and ESCRT proteins detected in these samples is shown below.

Sample	Gene name	Accession	Description	Coverage [%]	# Peptides	MW [kDa]
HCoV-OC43 VLP	N	Q4VID1	Nucleoprotein OS=Human coronavirus OC43	38	22	49.3
	M	Q4VID2	Membrane protein OS=Human coronavirus OC43	53	15	26.3
	TSG101	F5H442	Tumor susceptibility gene 101 protein OS=Homo sapiens	27	8	40.9
	PDCD6IP/ALIX	Q8WUM4	Programmed cell death 6-interacting protein OS=Homo sapiens	33	27	96
	VPS4A	Q9UN37	Vacuolar protein sorting-associated protein 4A OS=Homo sapiens	19	6	48.9
SARS-CoV-2 VLP	N	P0DTC9	Nucleoprotein OS=Severe acute respiratory syndrome coronavirus 2	80	41	45.6
	M	P0DTC5	Membrane protein OS=Severe acute respiratory syndrome coronavirus 2	34	19	25.1
	TSG101	F5H442	Tumor susceptibility gene 101 protein OS=Homo sapiens	32	11	40.9
	PDCD6IP/ALIX	Q8WUM4	Programmed cell death 6-interacting protein OS=Homo sapiens	47	45	96
	VPS4A	Q9UN37	Vacuolar protein sorting-associated protein 4A OS=Homo sapiens	42	19	48.9
	CHMP2A	M0R1T5	Charged multivesicular body protein 2a OS=Homo sapiens	28	10	29.6
	CHMP4B	Q9H444	Charged multivesicular body protein 4b OS=Homo sapiens	43	7	24.9
HCoV-OC43 virions	N	Q4VID1	Nucleoprotein OS=Human coronavirus OC43	63	36	49.3
	M	Q4VID2	Membrane protein OS=Human coronavirus OC43	63	19	26.3
	S	Q4VID5	Spike glycoprotein OS=Human coronavirus OC43	24	28	151.2
	HE	Q4VID6	Hemagglutinin-esterase OS=Human coronavirus OC43	20	11	47.5
	NSP2a	Q80872	Non-structural protein 2a OS=Human coronavirus OC43	72	23	32.2
	Protein I	Q4VID0	Protein I OS=Human coronavirus OC43	36	5	22.9
	pp1ab	P0C6X6	Replicase polyprotein 1ab OS=Human coronavirus OC43	6	35	797.4
	TSG101	F5H442	Tumor susceptibility gene 101 protein OS=Homo sapiens	19	7	40.9
	PDCD6IP/ALIX	Q8WUM4	Programmed cell death 6-interacting protein OS=Homo sapiens	37	27	96
	VPS4A	Q9UN37	Vacuolar protein sorting-associated protein 4A OS=Homo sapiens	24	10	48.9
Control sample	PDCD6IP/ALIX	Q8WUM4	Programmed cell death 6-interacting protein OS=Homo sapiens	7	7	96

Table S2. Summary of the roles of ESCRT proteins in coronavirus VLP production. The value represented that the relative VLP release ratio was calculated as the relative N level in culture supernatant divided by that in cell lysate. The release ratio of the VLP from control cells was set as 1.

	SARS-CoV-2	HCoV-OC43	MERS-CoV	HCoV-HKU1
Controli	1.00	1.00	1.00	1.00
TSG101i	0.55	0.59	0.62	0.64
VPS28i	0.59	0.51	0.61	0.68
MVB12Ai	0.15	0.04	0.18	0.43
MVB12Bi	0.84	0.49	0.83	0.76
VPS37Ai	0.67	0.93	1.01	0.86
VPS37Bi	0.47	0.85	0.66	0.63
VPS37Ci	0.70	0.45	0.65	0.59
CHMP1Ai	0.25	0.72	1.15	0.47
CHMP1Bi	1.49	1.01	0.96	0.66
CHMP2Ai	1.11	0.73	1.32	0.46
CHMP2Bi	1.37	0.46	1.19	0.71
CHMP3i	0.97	0.49	1.33	0.51
CHMP4Ai	1.23	0.68	1.53	0.77
CHMP4Bi	1.23	0.64	0.84	0.63
CHMP5i	0.99	0.46	0.84	0.33
CHMP6i	0.42	0.32	0.46	0.36
CHMP7i	1.11	0.90	1.56	0.60
ALIXi	1.37	0.74	1.10	0.40
VPS4Ai	0.16	0.07	0.23	0.23

Table S3. Sequences of siRNAs used in this study.

Target gene	siRNA sequence
Control siRNA	UUCUCCGAACGUGUCACGU
TSG101	CCUCCAGUCUUCUCUCGUCtt
VPS28	AGCCGGAGCUGUAUGAGGAAGUGAA
MVB12A	AGAAACGCAUGUGUGUGAAGCUGUU
MVB12B	GGAAAGACGGCUUAUUUAA
VPS37A	CGACAUCACUUAAUGGAUA
VPS37B	GCACGCUUGACCCAGAAAU
VPS37C	AGGCAUGAAGAUCGAAGAATT
CHMP1A	CCAAGAAGGCGGAGAAGGAtt
CHMP1B	UGGACAAAUUCGAGCACCAtt
CHMP2A	AGGCAGAGAUCAUGGAUAUtt
CHMP2B	GGAACAGAAUCGAGAGUUAtt
CHMP3 (1)	UGUGAAGAUUCCAGAGAUUtt
CHMP3 (2)	GGAAGAAGCAGAAAUGGAAtt
CHMP4A	AAGUAUGGGACCAAGAAUAtt
CHMP4B	CGAUAAAGUUGAUGAGUUAtt
CHMP5	AGAAUAUGGUCAAGCAGAAtt
CHMP6	GGAAAUGAGUGUCUGAACAtt
CHMP7	GGGAGAAGAUUGUGAAGUUtt
ALIX	GAACAAAUGCAGUGAUAUAtt
VPS4A	CCGAGAAGCUGAAGGAUUAtt

Table S4. Antibodies used in this study.

Antibodies	Source	Cat# No.
Mouse monoclonal Flag-specific antibody M2	Sigma-Aldrich	F3165
Rabbit polyclonal DYKDDDDK Tag antibody	Cell Signaling Technology	2368
Mouse monoclonal myc-specific antibody 9E10	Santa Cruz Biotechnology	SC-40
Rabbit monoclonal Myc-Tag antibody	Cell Signaling Technology	2278
Mouse monoclonal β-actin-specific antibody	Sigma-Aldrich	a5316
β-Actin Rabbit mAb	Abclonal	AC038
Mouse monoclonal bril specific antibody	This study	N/A
Mouse anti His-Tag mAb	Abclonal	AE003
Rabbit polyclonal SARS-CoV-2 Nucleocapsid specific Antibody	Sino Biological	40143-T62
Mouse monoclonal SARS-CoV-2 Membrane Protein specific antibody	This study	N/A
Mouse monoclonal SARS-CoV-2 Nucleocapsid Protein specific antibody	This study	N/A
Rabbit polyclonal Human coronavirus (HCoV-OC43) Nucleoprotein/NP specific Antibody	Sino Biological	40643-T62
Rabbit polyclonal MERS-CoV Membrane Protein specific antibody	Genetex	GTX134866
Rabbit polyclonal MERS-CoV Nucleoprotein Protein specific antibody	Genetex	GTX134868
Rabbit polyclonal Human coronavirus (HCoV-HKU1) Nucleoprotein/NP specific Antibody	Sino Biological	40642-T62;
Mouse monoclonal TSG101 specific antibody	Proteintech	67381-1-Ig
Rabbit polyclonal TSG101 specific Antibody	Proteintech	28283-1-AP
Rabbit polyclonal VPS28 specific Antibody	Proteintech	15478-1-AP
VPS28 Mouse Monoclonal Antibody	OriGene	TA505691
Rabbit polyclonal FAM125A(MVB12A) specific Antibody	Proteintech	25816-1-AP
Rabbit polyclonal MVB12B specific Antibody	Sigma-Aldrich	HPA043683
Rabbit polyclonal VPS37A specific Antibody	Proteintech	11870-1-AP
Rabbit polyclonal VPS37B specific Antibody	Proteintech	15653-1-AP
Rabbit monoclonal VPS37C specific Antibody	Abcam	ab151753
Rabbit polyclonal CHMP1A specific Antibody	Proteintech	15761-1-AP

Rabbit polyclonal CHMP1B specific Antibody	Proteintech	14639-1-AP
Rabbit polyclonal CHMP2A specific Antibody	Proteintech	10477-1-AP
Rabbit polyclonal CHMP2B specific Antibody	Proteintech	12527-1-AP
Rabbit polyclonal CHMP3 specific Antibody	Proteintech	15472-1-AP
Rabbit polyclonal CHMP4A specific Antibody	Sino Biological	204868-T44
Rabbit polyclonal CHMP4B specific Antibody	Proteintech	13683-1-AP
Rabbit polyclonal CHMP4C specific Antibody	OriGene	TA890004
Rabbit polyclonal CHMP5 specific Antibody	Sino Biological	201939-T44
Rabbit polyclonal CHMP6 specific Antibody	Proteintech	16278-1-AP8
Rabbit polyclonal CHMP7 specific Antibody	Proteintech	16424-1-AP
Rabbit polyclonal ALIX specific Antibody	Proteintech	12422-1-AP7
Rabbit polyclonal VPS4A specific Antibody	Proteintech	14272-1-AP
ABflo® 488-conjugated Goat anti-Rabbit IgG (H+L)	Abclonal	AS053
CoraLite® 594-conjugated TSG101 Monoclonal antibody	Proteintech	CL594-67381
Anti-Mouse IgG (whole molecule)-TRITC antibody	Sigma-Aldrich	T5393
mouse monoclonal p24-specific antibody P5F1	Liu et al., 2007	N/A
Anti-Mouse IgG (H+L), HRP Conjugate	Promega	w4021
Peroxidase-Conjugated Goat anti-Rabbit IgG(H+L)	ZSGB-Bio	ZB-2301

Data Set S1. The raw data corresponding to Table S1. Purified HCoV-OC43 VLP, SARS-CoV-2 VLP and HCoV-OC43 virions were subjected to MS analysis. The culture supernatant of the control cells without virion particles was purified through the same procedure to serve as a control (Control sample).
